# Tau-Cofactor Complexes as Building Blocks of Tau Fibrils

**DOI:** 10.3389/fnins.2019.01339

**Published:** 2019-12-13

**Authors:** Yann Fichou, Zachary R. Oberholtzer, Hoang Ngo, Chi-Yuan Cheng, Timothy J. Keller, Neil A. Eschmann, Songi Han

**Affiliations:** ^1^Department of Chemistry and Biochemistry, University of California, Santa Barbara, Santa Barbara, CA, United States; ^2^Department of Chemical Engineering, University of California, Santa Barbara, Santa Barbara, CA, United States

**Keywords:** tau protein, protein aggregation, tau oligomers, tauopathies, conformational transformation, aggregation seeding, cofactors

## Abstract

The aggregation of the human tau protein into neurofibrillary tangles is directly diagnostic of many neurodegenerative conditions termed tauopathies. The species, factors and events that are responsible for the initiation and propagation of tau aggregation are not clearly established, even in a simplified and artificial *in vitro* system. This motivates the mechanistic study of *in vitro* aggregation of recombinant tau from soluble to fibrillar forms, for which polyanionic cofactors are the most commonly used external inducer. In this study, we performed biophysical characterizations to unravel the mechanisms by which cofactors induce fibrillization. We first reinforce the idea that cofactors are the limiting factor to generate ThT-active tau fibrils, and establish that they act as templating reactant that trigger tau conformational rearrangement. We show that heparin has superior potency for recruiting monomeric tau into aggregation-competent species compared to any constituent intermediate or aggregate “seeds.” We show that tau and cofactors form intermediate complexes whose evolution toward ThT-active fibrils is tightly regulated by tau-cofactor interactions. Remarkably, it is possible to find mild cofactors that complex with tau without forming ThT-active species, except when an external catalyst (e.g., a seed) is provided to overcome the energy barrier. In a cellular context, we propose the idea that tau could associate with cofactors to form a metastable complex that remains “inert” and reversible, until encountering a relevant seed that can trigger an irreversible transition to β-sheet containing species.

## Introduction

The intrinsically disordered human tau protein has the primary function of stabilizing microtubules in healthy individuals, whereas under pathological conditions, soluble tau dissociates from microtubules and aggregates into distinct fibrillar constructs (Goedert et al., 2006). The formation of neurofibrillary tangles from human tau has been verified to be directly part of the diagnosis of Alzheimer’s disease and other neurodegenerative diseases including chronic traumatic encephalopathy (CTE) and corticobasal degeneration (CBD), collectively referred to as tauopathies (Lee et al., 2001). However, viable therapeutic approaches do not currently exist to alter the course of such diseases. Tau is believed to be capable of spreading aggregate pathology through interconnected neuronal pathways *in vivo* in a “prion”-like process (Sanders et al., 2014; Stancu et al., 2015; Kaufman et al., 2016; Woerman et al., 2016), making the elucidation of intermediate aggregation species and seeding mechanisms a top priority in the study of the pathological tau aggregation pathway. At the current state of understanding, it is unclear what the defining molecular-level feature of aggregation-prone and seeding-competent tau is, even in a simplified *in vitro* system. The ultimate question in the prion hypothesis is what factor is responsible for converting normal to pathological tau.

Tau protein is highly charged, overall positively, hydrophilic and therefore very stable in aqueous conditions across a large range of pH, temperatures and concentrations. Tau aggregation *in vitro* is most commonly triggered by external inducers or cofactors, such as heparin (Goedert et al., 1996), RNA (Kampers et al., 1996) or arachidonic acid (Wilson and Binder, 1997). The use of cofactors to make and study tau aggregation *in vitro* has two main origins. First, it represents a convenient, reproducible procedure to form tau fibrils in order to mechanistically study the process of amyloid formation. Second, several cofactors have been colocalized with tau neurofibrillary tangles in brain tissue (Goedert et al., 1996; Ginsberg et al., 1997), providing a biological justification for these cofactors. Recently, tau fibrils induced by addition of heparin, the most commonly used cofactor, were found to be heterogeneous and different from brain-extracted fibril structures (Fichou et al., 2018b; Zhang et al., 2019a), thereby questioning the quality of cofactor-induced fibrils as an aggregate model. However, recent studies demonstrated the biological relevance of cofactors in tau aggregation, by indirectly showing its involvement in seeding and fibril stability (Fichou et al., 2018a) and by showing that a cofactor of unknown identity is part of the fibril core in CTE (Falcon et al., 2019) and CBD (Zhang et al., 2019b). Through the study of cofactor-induced aggregation of recombinant tau *in vitro*, we endeavor to find defining molecular features of aggregation-prone or seeding-competent tau species.

At a mechanistic level, how cofactors interact with tau to trigger aggregation is not well understood. Heparin-induced tau aggregation has been modeled in several quantitative studies (Ramachandran and Udgaonkar, 2011; Shammas et al., 2015; Kjaergaard et al., 2018) and has provided a global picture where heparin interacts with tau to form aggregation competent oligomers that evolve to fibrils. However, even a property as basic as the inclusion of heparin into the mature fibrils is disputed, with multiple conflicting reports saying that heparin is either not part of fibrils (von Bergen et al., 2006; Carlson et al., 2007; Ramachandran and Udgaonkar, 2011) or part of the final aggregate (Sibille et al., 2006; Dinkel et al., 2015; Fichou et al., 2018a). It is perhaps due to these observations that heparin’s treatment in literature has largely been that of a useful catalyst rather than a critical reactant. In this study, we used heparin and RNA as cofactors to understand the conformational evolution of tau, the population of intermediate species, as well as the role of the cofactors in assisting the seeding of aggregation to further refine the tau-aggregation model.

A previous study of ours used electron paramagnetic resonance (EPR) spectroscopy to reveal experimentally the existence of disordered intermediate aggregates (termed I) of tau lacking β-sheet structure (Pavlova et al., 2016). Additionally, we have identified aggregation-prone soluble tau species with an extended conformation around PHF6^(*)^ (termed S^∗^), distinct from the pre-heparin-activated tau species which are composed of a more compact conformation around PHF6^(*)^ (termed S) (Eschmann et al., 2017). The key unresolved question is what are the seeding competent species or factors that are responsible for inducing, as well as propagating aggregation? With that in mind, we ask: (1) Do the disordered aggregation intermediates (I) quantitatively convert *on pathway* to mature fibrils? (2) What is the role of heparin in populating the intermediate stages of tau, namely S^∗^, I, and mature fibrils? And (3) should cofactors be considered a constitutive part of tau fibrils?

**FIGURE 1:**
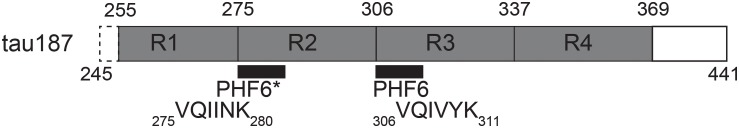
Schematic of tau187. It contains all four repeat domains R1-R4, minus 10 residues of R1, and the C-terminus. The N-terminal end contains a six histidine tag for purification (not shown).

In order to accomplish these goals, we employed a combination of EPR spectroscopy tools together with multiple biochemical assays which allow for the direct observation and characterization of cofactor-induced aggregation intermediates. We used a truncated version of tau, containing the four repeat regions as well as the C-terminus (residues 255–441 of full length 2N4R), termed tau187 (Figure 1). Continuous wave (cw) EPR lineshape analysis was employed to report on spin-label mobility and β-sheet formation as well as to quantify intermediate species, as previously demonstrated (Pavlova et al., 2016). Intra-protein distances and conformational rearrangement around PHF6^∗^ (^275^VQIINK^280^) were quantified by applying Double Electron-Electron Resonance (DEER) spectroscopy to tau187 doubly labeled with MTSL at sites 272 and 285. We also assessed time-resolved local dehydration around selected tau187 sites by Electron Spin-Echo Envelope Modulation (ESEEM) measurements. This report makes the case that cofactors are active parts of the tau aggregation process and should not be considered as a mere catalyst.

## Results

### Heparin Stoichiometry Dictates Tau Population Embedded in Fibrils

**FIGURE 2:**
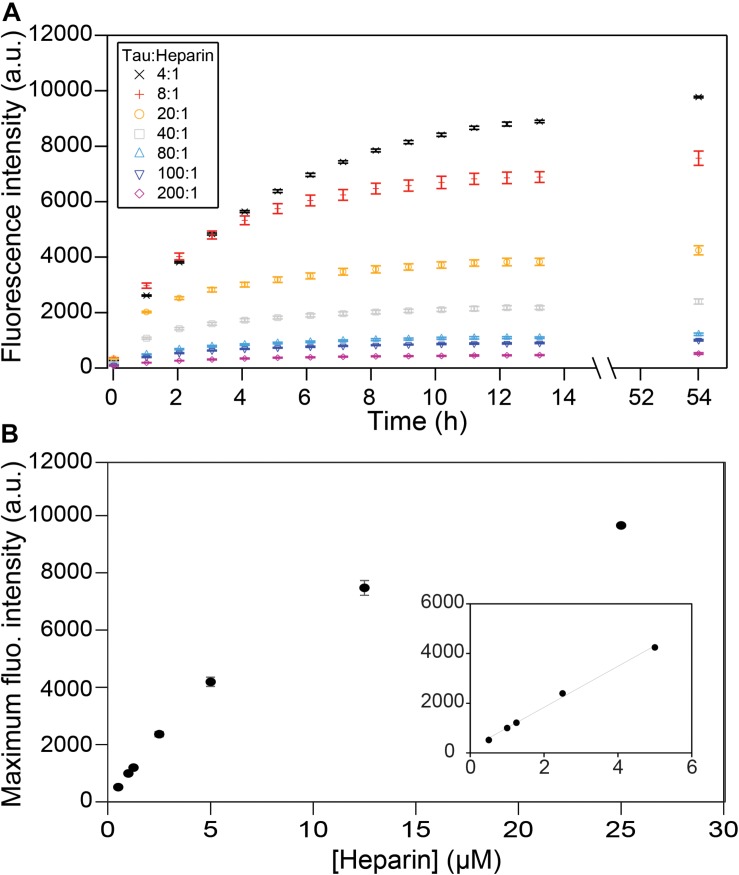
ThT fluorescence measuring β-sheet formation as a function of time and tau:heparin molar ratio. Tau concentration is 100 μM. Kinetic curves **(A)** have plateaued after 53 h and the fluorescence intensities at this time are reported as a function of heparin concentration **(B)**. A zoom at low heparin concentration (**B**, inset) shows a linear behavior. Gaps between 13 and 54 h represent reading interruptions. Error bars represent standard deviation over 3 repeats.

We first investigated the relation between the quantity of heparin incubated with tau and the fibril quantity formed at equilibrium. Tau187 contains two cysteines, one of which was mutated to serine (C291S) so that only one cysteine (C322) could be spin-labeled for cw-EPR experiments (see subsequent sections). This construct is referred to as tau187 throughout the manuscript. Fluorescence kinetic measurements were carried out using thioflavin T (ThT), a fluorescent dye that has been established to bind specifically to β-sheet structures of amyloid proteins (Nilsson, 2004; Biancalana and Koide, 2010) and thus allowed the determination of the total tau embedded in β-sheet structures. Increase in ThT fluorescence intensity occurs rapidly after heparin addition to spin-labeled tau187, where no extended lag phase is observed (Figure 2A). These measurements were performed with spin-labeled tau187 so that the kinetics could be directly compared with cw-EPR experiments, but the same results were found with unlabeled tau187 (Supplementary Figure S1). The ThT fluorescence time course was measured out to 54 h; the results showed that the total β-sheet content does not converge to the same value between samples that contained equal quantities of tau incubated with different quantities of heparin. In fact, the maximum ThT value was found to scale linearly with heparin at low concentrations (Figure 2B), but not at higher concentrations, possibly due to the formation of off-pathway oligomers (Ramachandran and Udgaonkar, 2011). In addition, increasing the amount of fibril ends by sonication (Supplementary Figures S2, S3) or introduction of intermediates (Supplementary Figure S4) did not change the ThT intensity. This result demonstrates that the systematic decrease in β-sheet content seen with decreasing amount of heparin (and unchanged tau content) is not a kinetics driven effect. Rather, the data shows that heparin dictates and limits the quantity of tau transforming into β-sheets at equilibrium, suggesting that heparin is an obligatory reactant of tau187 fibril formation that does not free up even after fibrils are formed.

### Heparin Stoichiometry Dictates the Conversion to Aggregation-Prone Conformations

**FIGURE 3:**
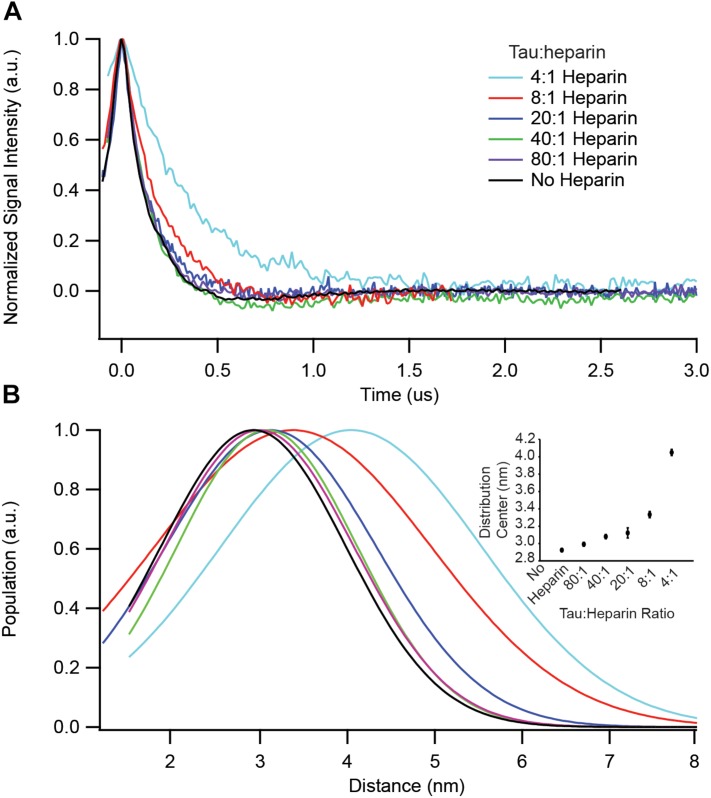
DEER spectroscopy of doubly labeled tau187 at positions 272 and 285. Data points shown are for before (black) and 1 h after addition of the following stoichiometries: 4:1 (cyan), 8:1 (red), 20:1 (blue), 40:1 (green), and 80:1 (purple) tau:heparin molar ratio. **(A)** Background subtracted and normalized DEER time-domain signal. **(B)** Intramolecular distance distribution assuming a Gaussian distribution. The inset shows the Gaussian distribution center as a function of heparin amount. Error bars are estimated from the covariance matrix of the fitting procedure.

We have identified key structural transformations that tau undergoes during aggregation (Eschmann et al., 2017), however, it is unclear what role heparin plays in these transformation processes. Does heparin facilitate only the formation of aggregation-driving tau species or is it directly involved in each structural transition? We examined the dependence of the S^∗^ population, the aggregation-prone conformation of tau that has been identified in an earlier report (Eschmann et al., 2017), on heparin concentration. For that, we used DEER measurements on doubly labeled tau187, at position 272 and 285, to probe the opening of the segments flanking the PHF6^∗^ region. In order to ensure that only intra-tau, not inter-tau, distances are measured, spin dilution was employed by mixing MTSL and dMTSL – the diamagnetic analog of MTSL – labeled tau187 at a 1:10 molar ratio (Fichou et al., 2017). At a 4:1 tau:heparin stoichiometry, tau187 is maximally extended at a distance of ∼4.1 nm, as shown in Figure 3. However, when adding heparin at 5-, 10- or 20-fold dilution (20:1, 40:1, and 80:1 tau:heparin, respectively), we observed no clear conformational extension of the 272–285 tau segment (Figure 3) while a twofold dilution (8:1) invoked a small extension around PHF6^∗^. Thus, a fivefold reduction in heparin concentration, i.e., the presence of a sub-stoichiometric amount of heparin (tau:heparin 20:1), diminishes the S^∗^ population to below our measurement threshold, indicating the direct dependence of the S^∗^ population on the tau:heparin stoichiometry. In other words, heparin is necessary to trigger and maintain the conformational rearrangement of S to S^∗^ that precedes aggregation. In addition, we spin labeled heparin and measured the cw-EPR lineshape before and after incubation with tau187. The lineshape broadened (most likely due to weak spin exchange or/and lower correlation time) upon addition of tau187 (Supplementary Figure S5), revealing a direct and persistent interaction of heparin and tau187. More specifically, the line broadening already occurs within 30 min after incubation, showing that heparin is directly involved in the S to S^∗^ transition and intermediate formation measured within 1 h of incubation (Figure 3). This result suggests that heparin acts as a templating reactant that triggers the conformational rearrangements necessary for aggregation, in good agreement with a previous report showing that heparin directly participates in the formation of early intermediates (Kjaergaard et al., 2018).

### Aggregation Involves the Formation of On-Pathway Oligomers

We sought to gain information on the mechanisms of cofactor-induced aggregation, by measuring the formation of intermediate species along the aggregation pathway. A distinct single line component of the EPR spectra was first recognized and utilized by Margittai and Langen (2004) as a diagnostic tool to characterize parallel β-sheet structures for the study of tau fibrils. Our group has used cw-EPR to quantify the populations of tau molecules that (i) participate in a cross-β sheet arrangement (termed F) giving rise to so-called spin-exchange interaction between the spin labels, (ii) participate in oligomer/aggregate intermediates (termed I) that do not form in-register cross-β sheets but are still characterized by a slow rotational time, and (iii) remains monomeric (termed M) and therefore mobile (Pavlova et al., 2016). Systematically deconvoluting the different contributions to the EPR lineshape allowed for the tracking of the formation and transformation of these tau species as a function of aggregation time.

**FIGURE 4:**
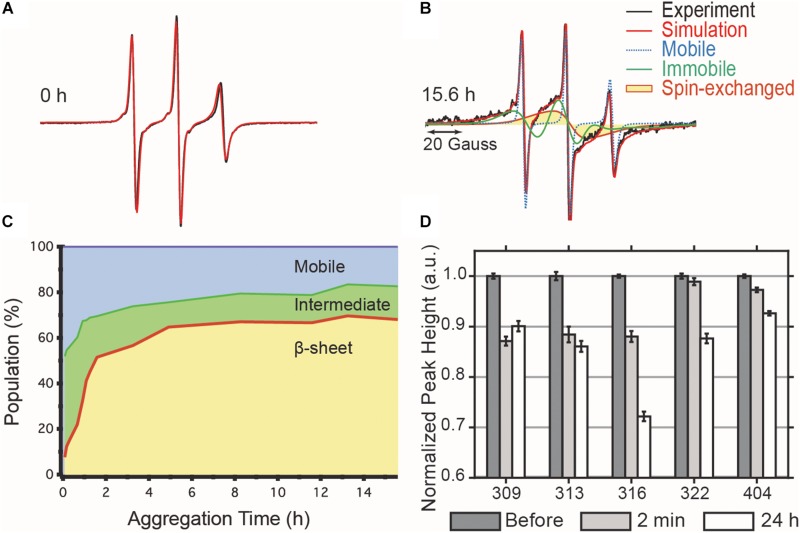
cw-EPR time course of 4:1 tau:heparin molar ratio induced aggregation. **(A)** Before heparin addition, the EPR line shape (black) can be simulated by a single mobile component (red). **(B)** 15.6 h after heparin addition, the spectrum is broadened and the simulation requires the addition of an immobile (green) and spin-exchange (yellow) component in order to obtain a good fit. **(C)** Time evolution of the relative contributions of each component. **(D)** ESEEM relative deuterium peak normalized by the value before heparin addition and plotted versus MTSL-labeled residue. The decrease in peak height, reflecting a local dehydration, at 2 min shows that the arrangement of the intermediates is consistent with the structural features of matures fibrils. Error bars represent the standard deviation of the baseline noise.

We tracked the time course of the population and turnover of monomeric (M), intermediate (I) and cross-β-rich (F) species of tau187 (Figure 4). Prior to heparin addition, the EPR lineshape is 100% mobile (i.e., population of M is 100%) and displays a narrow nitroxide EPR spectrum (Figure 4A). After aggregation, the EPR lineshape is broadened, and contributions from both a slow component (termed immobile) originating from non-amyloid aggregates and a single line spin-exchange component, originating from cross-β structures, are required to simulate the EPR spectra, as shown for the EPR spectra of tau after 15.6 h upon heparin addition (Figure 4B). The fit and the raw spectra are overlaid for each time point in Supplementary Figure S6. Time evolution of the populations of the different components is summarized in Figure 4C. Within 5 min of heparin addition, 44% of the tau population forms I, while the population of tau incorporated in β-sheet F remains small (<10%). As aggregation progresses, the intermediate species that constituted up to ∼40% of the total population convert into the F species, which constitute a majority of tau (65%) after 5 h of aggregation. The quantitative conversion of the intermediate population into β-sheet containing species demonstrates that the intermediates are on-pathway to fibrils. The remaining population of the intermediate component after 5 h (less than 15%) might originate from molecules either composing the end of tau fibrils (also previously referred to as interfacial tau; Pavlova et al., 2016) or forming off-pathway intermediates. Interestingly, the ThT-derived measurement of β-sheet structured species (Figure 2A, maximum reached after 15 h) systematically lagged behind the populations as quantified by EPR lineshape analysis (Figure 4C, maximum reached at about 5 h), i.e., the formation of β-sheet as revealed by EPR occurred more rapidly than captured by ThT fluorescence measurements. The ThT intensity may depend on a threshold aggregate size in contrast to EPR that measures the spin-exchange component as soon as two spin labels come within close proximity.

The dynamic intermediates, I, lack measurable β-sheet structure in the R3 region according to their EPR lineshape (i.e., no spin-exchange), but the question is whether they are deficient of any residual structural features. In other words, does the formation of I occur by structurally directed transitions or by non-specific collapse? We applied ESEEM spectroscopy to measure relative changes in the local water density on the protein surface within a ∼3–6 Å shell of a site-specific spin label over the course of aggregation (Volkov et al., 2009). In essence, ESEEM spectroscopy counts the number of deuterated water nuclei around the spin label, where the intensity of the D^2^ peak is proportional to the number of these nuclei. We carried out ESEEM spectroscopy with singly MTSL labeled tau187 at several sites within the R3 region (residues 309, 313, 316, 322) and in the C-terminus (residue 404). Decrease in the D^2^ peak height, reflecting a decrease in water density, is observed within 2 min after heparin addition for all sites (Figure 4D). A gradient of dehydration is observed where sites 309, 313, and 316 exhibit the strongest effect and dehydration becomes progressively less visible for the sites close (322) or in the C-terminal region (404) (Figure 4D). After 24 h of aggregation, site 309 experiences a slight recovery in the D^2^ density, while the D^2^ densities around sites 313, 316, 322, and 404 are systematically decreased. The timing of the initial dehydration (i.e., within 2 min) clearly precedes the development of the amyloid structure seen by EPR lineshape analysis, but it is within the development time scale of the I intermediates (Figure 4C). Thus, ESEEM suggests that I species structures are *en route* to aggregates; dehydration is stronger around residues that are expected to be buried in the mature fibrils (Margittai and Langen, 2006; Zhang et al., 2019a).

### A Well-Defined Cofactor-Tau Interaction Pattern Is Necessary to Form Fibrils

**FIGURE 5:**
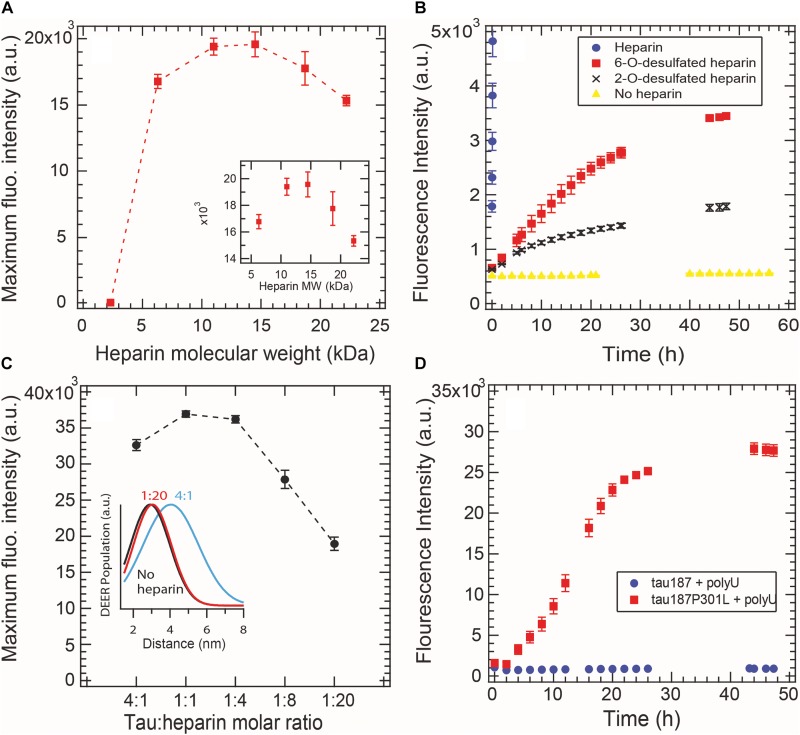
Cofactor length, stoichiometry, charge state and nature modulate tau187 aggregation, as probed by ThT fluorescence. ThT fluorescence was measured over time and the maximum plateau value is plotted for **(A)** different heparin lengths (constant mass ratio) and **(C)** different tau:heparin molar ratios. DEER distance distribution between residues 272 and 285 (inset **C**) shows that S^∗^ is not present at 1:20 tau:heparin ratio, **(B)** Tau187 was incubated with fully sulfated (purple circle), 6-O desulfated (red square), 2-O desulfated (black cross) heparin or no heparin (yellow triangle) at 4:1 tau:heparin ratio. The sulfated heparin curve converges to about 3.8 × 10^4^ fluorescence units and was cut to better highlight the difference between 6OD and 2OD. **(D)** PolyU (500 μg/mL) incubated with tau187 and tau187-P301L. The protein concentration was 50 μM in all panels. Gaps in the ThT kinetics **(B,D)** originate from reading interruptions. Error bars represent standard deviation over 3 repeats.

Aggregation-promoting cofactors, such as heparin or RNA, are usually described as polyanions that somewhat screen the positively charged tau repeat domain (Sibille et al., 2006). We investigated whether this simple charge compensation picture can sufficiently describe cofactor-induced aggregation by varying the nature and length of the cofactors. The heparin typically used in the literature, and elsewhere in this study, is polydisperse as it is directly extracted from porcine intestinal mucosa. Here we used purified monodisperse heparins of various molecular masses to trigger tau aggregation, quantified by ThT fluorescence. For all heparins, a constant tau:heparin mass ratio of 4:1 was used. Figure 5A reports the maximum ThT fluorescence reading of fully grown fibrils formed with heparins of different molecular weights. Heparins below 2.3 kDa are incapable of forming tau fibrils. The maximal ThT value increased with heparin’s molecular weight until reaching a maximum between 11 and 15 kDa. Addition of higher molecular weight heparin (18 and 22 kDa) decreased the maximum ThT intensity, suggesting an optimal heparin length of 11–15 kDa to promote fibril formation of tau187.

We furthermore looked at the influence of the charges on heparin molecules. Heparin is a highly sulfated glycosaminoglycan that mostly possesses 4 charges per disaccharide (3 sulfate and 1 carboxyl groups). We compared the aggregation triggering propensity of heparin, 2-O desulfated (2OD) and 6-O desulfated (6OD) heparin (Figure 5B). Desulfation of either of the two sites resulted in a loss of ThT signal of about one order of magnitude, consistent with the picture that charge interaction is critical for the ability of heparin to trigger aggregation. However, when comparing the aggregation propensity of the two desulfated heparins, we observed that, despite the same charge density, 6OD heparin gives rise to a significantly larger ThT fluorescence signal than 2OD heparin. This modulation of the ThT intensity could originate either from a change in the total amount of fibrils (i.e., 6OD heparin is more potent to induce tau fibrils) or from a change in the fibril structure that would react differently with ThT. Interestingly, this modulation of ThT intensity triggered by the different heparin (ThT_heparin_ > ThT_60__D_ > ThT_2__OD_) cannot be explained by a modulation of tau-heparin affinity, as heparin and 2OD heparin have a similar affinity to tau, while 6OD has a drastically lower affinity (Zhao et al., 2017).

In Figure 5C, we added excess heparin at a constant tau concentration beyond the 4:1 tau:heparin molar ratio typically used. The data showed that increasing the quantity of heparin, above an optimal ratio of about 1:1 tau:heparin in our conditions, decreased the maximum ThT intensity. This result shows that a defined tau:heparin molar stoichiometry is necessary to trigger optimal aggregation, as previously observed (Carlson et al., 2007; Ramachandran and Udgaonkar, 2011). DEER measurements around PHF6^∗^ further confirmed this results by showing the quasi absence of the aggregation-prone open conformation S^∗^ at 1:20 tau:heparin (Figure 5C inset and Supplementary Figure S7).

RNA molecules are other polyanions that have been reported to be capable of triggering tau aggregation (Kampers et al., 1996). Here we tested the efficacy of polyU RNA in aggregating tau187 with and without the disease-associated mutation P301L (Figure 5D). In contrast to heparin, polyU does not spontaneously trigger aggregation of tau187 over the time course of 26 h. However, a single-point mutation P301L in tau187, which does not add any charge to tau, led to polyU-induced aggregation, as shown by ThT (Figure 5D) and TEM (Supplementary Figure S8). Note that the kinetics of polyU-induced tau aggregation is different from heparin-induced aggregation (compare Figure 5D with Figure 2A), potentially revealing different aggregation pathways. The P301L mutation was recently proposed to promote aggregation by increasing the population of aggregation-prone conformers (Chen et al., 2019). The data here suggests that this conformational rearrangement in combination with the presence of polyU is highly favorable for aggregation. Note that neither tau187 nor tau187-P301L aggregate spontaneously without polyU (Supplementary Figure S9).

These results highlight that the simple view of a charge compensation mechanisms, in which the basic tau repeat domains are allowed to self-assemble by adding anions, is not sufficient. Rather, a well-defined pattern of interaction between tau and its cofactor seems to be required, which is influenced by the length, charge state, steric environment, and stoichiometry of the cofactor, as well as the conformational landscape of the protein.

### Tau-Cofactor Form a Complex That May Not Spontaneously Evolve to Fibrils

**FIGURE 6:**
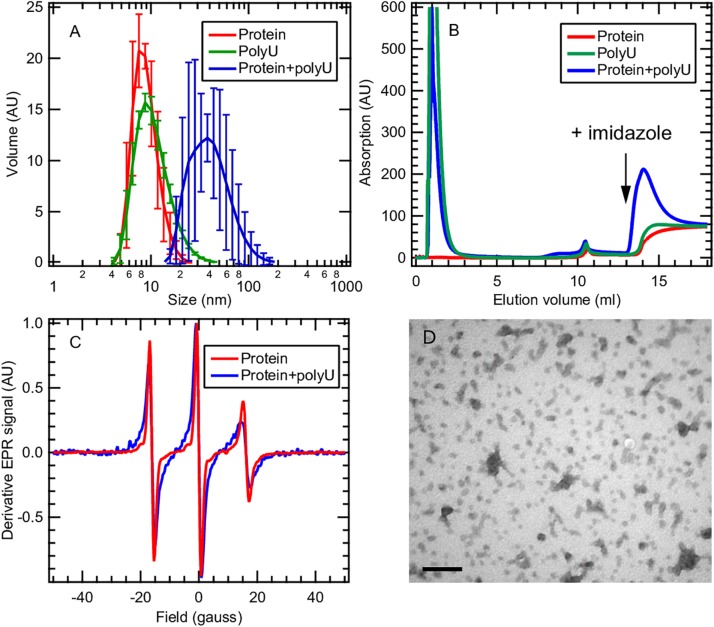
Tau187 and polyU form a complex. **(A)** tau187 (100 μM) incubated with polyU (1 mg/mL) (blue) exhibit a larger hydrodynamic radius than tau187 alone (red) or polyU alone (green), as shown by DLS. Error bars represent standard deviation over 3 independant repeats, each composed of 3 measurements of the same sample. **(B)** tau187, which possesses a histidine tag, can be immobilized onto a Ni-affinity column (mounted on a FPLC system), and eluted by imidazole. The absorption value reports on the presence of polyU, which has an absorption coefficient 100 times larger than that of tau187. The fact that RNA is eluted with imidazole when incubated with tau187 (blue trace) shows that they form a complex that is immobilized in the Ni-column. **(C)** The presence of the complex is further confirmed by cw-EPR applied on tau187 labeled at position 322. The lineshape broadening observed when tau187 is incubated with polyU (blue) reflects a slower correlation time and therefore shows that tau187 is no longer monomeric. **(D)** TEM picture of tau187 incubated with polyU for 10 min. Scale bar is 200 nm.

Here we focus on the tau187 + polyU RNA system that evolves into fibrils only in the presence of the P301L mutation (Figure 5D). This observation raises the question of how a single-point mutation can completely enable/disable RNA-induced aggregation. We applied multiple techniques to characterize the tau187 + polyU sample, within 20 min after co-incubation, by dynamic light scattering (DLS), cw-EPR lineshape analysis and affinity chromatography. DLS was measured on tau187 alone, polyU alone and a mixture of both (Figure 6A). We observed a significant increase of hydrodynamic radius (Rh) when the compounds were mixed together to a value of about 40 nm. This Rh is compatible with a small complex containing several molecules, but given the uncertainty on the complex structure, we cannot deduce the exact stoichiometric composition from the Rh. The presence of tau87-polyU complexes was confirmed by electron microscopy (Figure 6D) showing oligomers of few tens of nm, in good agreement with DLS data. Interestingly, round-like oligomers of about 30 nm seems to agglomerate in elongated assemblies that might represent prefibrillar assemblies. Note that these oligomers do not originate from liquid-liquid phase separation (LLPS) as they do not appear to merge or be perfectly round. In addition, the tau187:polyU ratio used does not fulfill the charge balance requirement to form LLPS (Lin et al., 2019) (excess of negative charges from RNA). The formation of this complex was further confirmed by cw-EPR that showed a lineshape broadening upon addition of polyU to tau187 (Figure 6C). This broadened linshape, which reflects a slowdown of the spin label dynamics, could be fit with a single component (Supplementary Figure S10), suggesting that most of the tau molecules are part of a complex. The best fit was obtained utilizing a rotational correlation time of 1.3 ns, which is lower than the one found for the spin labeled part of intermediate species identified in heparin-induced aggregation (∼3.4 ns, see Figure 4 and Supplementary Table S1). This difference suggests that the polyU-tau187 complex is smaller than the heparin-induced intermediate species identified in Figure 4. Similar lineshape broadening was observed when tau187 was labeled at a different position (residue 272; Supplementary Figure S11), suggesting that this decrease in dynamics is not a local effect but reflects the global property of the protein.

We furthermore demonstrated the presence of this complex by affinity chromatography. Tau187 was expressed with a poly-histidine tag that can be immobilized onto a Ni-affinity column. We loaded either tau187 (0.4 mg), tau187 + polyU (0.4 mg + 0.4 mg, respectively) or polyU (0.4 mg) onto the Ni-affinity column connected to a FPLC system and followed the absorption at 280 nm (Figure 6B). Note that the absorption at 280 nm is 100-fold lower for tau187 than for polyU (about 0.13 and 11 mg^–1^.mL.cm^–1^ for tau187 and polyU, respectively), so that it reports on the presence of polyU. The sample was injected at V = 0 mL and the peaks visible at 1–2 mL correspond to the material flowing through the column without interaction. After flowing buffer, imidazole was injected at V = 13 mL to elute the material that was immobilized in the column. Because of the low absorption of tau187, it is essentially invisible from the chromatogram (red trace; the step at 14 mL corresponds to imidazole absorption; Supplementary Figure S12). As expected, polyU flows entirely through the column and none of it is eluted with imidazole (green trace). However, when tau187 mixed with polyU are injected, a strong polyU peak is eluted with imidazole. We confirmed that this peak contained both the protein and RNA by running SDS-page gel and acquiring the absorption spectrum (Supplementary Figure S12). The elution of polyU from the affinity column (i) demonstrates the existence of a tau187-polyU complex that can be immobilized onto an affinity column via the histidine tag present on the protein and (ii) offers a reliable way to purify this complex by collecting the elution peak. After collection of the elution peak and removal of the imidazole by buffer exchange, we were able to measure the polyU concentration by UV absorption and the protein concentration by spin counting (see section Materials and Methods), which revealed a polyU:tau187 mass ratio of ∼2:1. We also showed that this complex could be destabilized either by increasing salt concentration in the buffer (Supplementary Figure S13) or by digestion of polyU RNA with RNase A (Supplementary Figure S14). These results demonstrate that tau187 mixed with polyU form an electrostatically stabilized complex that does not spontaneously aggregate to fibrils. The existence of this ThT-inactive polyU-tau complex was also shown for other tau187 mutants (with zero and two native cysteines) and for the full length tau 2N4R isoform (Supplementary Figure S15), as well as for a different buffer (Supplementary Figure S16) and across a wide range of concentration (Supplementary Figure S17).

### Tau-Cofactor Complex Is Metastable and Form Fibril Building Blocks

**FIGURE 7:**
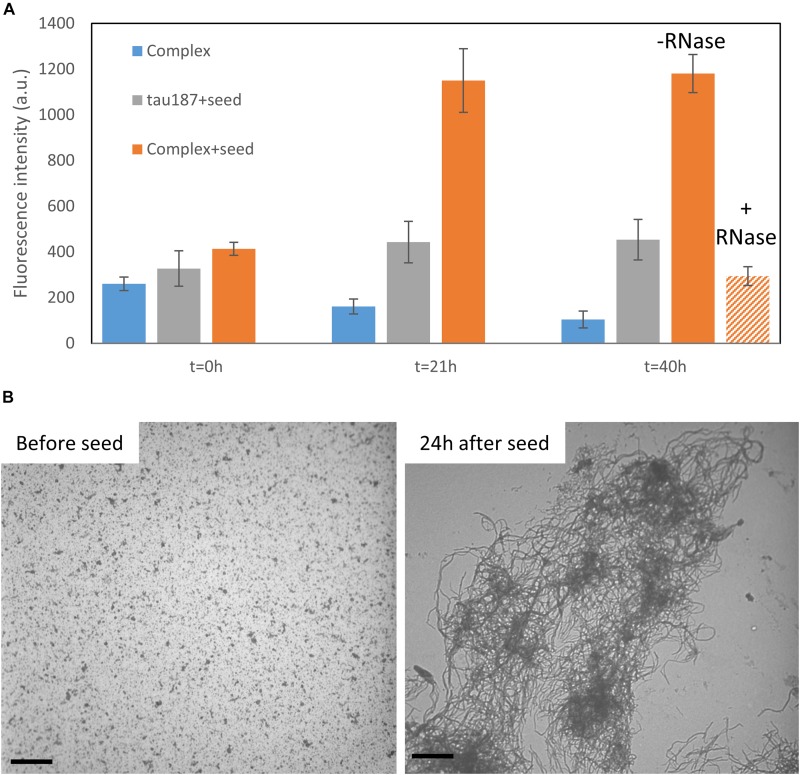
Tau187-polyU complex is stable but can be converted to ThT active species in the presence of a seed. **(A)** ThT intensity of the complex sample eluted from a Ni-affinity column with (orange) or without 5% seed (blue). In comparison, fresh tau187 is incubated with seed (gray). The seed is added at time *t* = 0 h and the ThT value, reported at 21 h, increased significantly for the seeded complex. RNase is added right after 21 h and significantly reduces the ThT intensity. Full kinetics curves are shown in Supplementary Figure S18. **(B)** TEM pictures show tau187-polyU complexes (without Ni-affinity purification) that are converted to fibrillary assemblies 24 h after seed addition. Scale bars are 1 μm.

We then asked the question whether this complex that appears stable at room temperature could be converted to fibrils. We first mixed tau187 and polyU and purified the complex by affinity chromatography (see previous section). The elution peak was collected and buffer exchanged to remove imidazole. This purified complex was incubated with 5% (protein mass ratio) of mature heparin-induced fibrils as seeds, while monitoring ThT fluorescence (Figure 7A and Supplementary Figure S18). The ThT intensity significantly increased when seeds were added to the complex, but not when added to the protein alone (i.e., not complexed with polyU), showing that the tau-RNA complex can be efficiently seeded to form ThT-active species. The seed-enabled conversion from oligomer to amyloid fibrils was confirmed by TEM showing that oligomers before seed addition (10 min incubation of polyU + tau187) turned into fibrillary assemblies 24 h after seed addition (Figure 7B). Once the ThT signal was maximal and steady (21 h), RNAse A was added to the seeded fibrils, resulting in a significant loss of the ThT signal (Figure 7A). This is consistent with previous results (Fichou et al., 2018a) showing that mature cofactor-induced tau fibrils depolymerize upon digestion of the cofactor. The full ThT intensity curves are provided in Supplementary Figure S18. These results show that (i) tau187-polyU complex is metastable and can be converted to fibrils given that an appropriate trigger is provided (seed) and (ii) the entire complex (not just tau) is a constituent of the fibrils as removal of the cofactor results in the destabilization of the fibrils.

## Discussion

In this report, we first reinforced previous findings showing that (i) the amount of tau aggregates formed by cofactor induction is limited by the stoichiometry of the cofactor relative to tau (Carlson et al., 2007; Ramachandran and Udgaonkar, 2011) and that (ii) tau aggregation to fibril is preceded by the formation of on-pathway intermediates (Ramachandran and Udgaonkar, 2011; Shammas et al., 2015; Kjaergaard et al., 2018). The latter intermediates are directly measured and quantified by EPR lineshape analysis. We furthermore showed that the molar stoichiometry of the cofactor relative to tau dictates the extent of conversion toward aggregation-prone tau conformers (either present as monomers or as part of intermediate species), recalling a chaperone-like activity of the cofactor, but one in which the cofactor remains associated with tau. We highlighted that seemingly relatively small modifications in the polyanionic cofactor, such as changing the charge location, or a single point mutation of tau, can drastically change the aggregation propensity of tau. Remarkably, we found that RNA and tau187 associate to form a soluble complex, apparently stable as it does not spontaneously trigger fibril formation of tau. However, we showed that this complex is metastable and can be converted to fibrils when exposed to a seed.

The importance of the cofactor:tau stoichiometry was studied in great detail by Carlson et al. (2007) as well as Ramachandran and Udgaonkar (2011). Similar to what is reported here, they found the existence of a cofactor-limited regime at low cofactor concentrations, and an inhibitory regime at high cofactor concentration, where addition of cofactor inhibited fibril formation. They postulated that the cofactor converts tau into an aggregation-prone form, acting as an allosteric regulator (Carlson et al., 2007), or into an aggregation-prone intermediate (Ramachandran and Udgaonkar, 2011). However, because they could not detect the cofactors as part of the mature fibrils, the concept of heparin as a catalyst was still compatible with these studies, leaving an unresolved question open as to why the cofactor could not be used multiple times to convert all available tau. The finding here (Figure 7A) and elsewhere (Fichou et al., 2018a) that removal of the cofactor by enzymatic digestion destabilizes the mature fibrils uncovers the missing piece of evidence to claim that cofactors are critical reactants of the tau-cofactor aggregation reaction. In other words, we show that the entire tau187-polyU complex (not just tau) is a constituent of the fibrils as removal of the cofactor results in the destabilization of the fibrils. Strikingly, the structure of tau aggregates present in corticobasal degeneration was solved very recently (Zhang et al., 2019b) and highlighted the incorporation of an unknown polyanionic cofactor in the fibril core. It is important to note that these findings do not exclude the existence of other aggregation pathways that do not involve cofactors. Such pathways could be promoted by specific post-translational modifications (PTMs) (Despres et al., 2017) or fragmentations (Al-Hilaly et al., 2017).

We also found that the population of tau transforming from S (tau adopting a compact conformation around PHF6^∗^) to S^∗^ (tau adopting an extended conformation around PHF6^∗^) (Figure 3) depends on the heparin concentration, showing that heparin is directly involved in the structural conversion of tau and acts as a templating reactant. Interestingly, when adding an excess amount of heparin (20:1 heparin:tau), this conversion to S^∗^ conformations is no longer observed, in good agreement with the low maximum ThT signal (Figure 5C). This shows that the stoichiometry between tau and cofactors is critical for the formation of S^∗^. Ramachandran and Udgaonkar (2011) modeled the decrease in ThT signal observed when increasing heparin amount with off-pathway oligomers that have excess of heparin. Our results show that these off-pathway oligomers are lacking the aggregation-prone conformations S^∗^.

We found that, at equal quantity of a heparin cofactor, the length of heparin monomers influences the final population of β-sheet structured species. This finding suggests that heparin templates aggregation via a well-defined structural change that requires an optimal polyanion chain length. Recall that the same tau:heparin mass ratio is compared to ensure the same quantity of saccharide units are present in each experiment. The observation that there is a lower molecular weight cutoff for heparin below which it cannot induce aggregation suggests that a polymer chain of heparin is necessary, not merely the heparin monomers, to trigger aggregation. If we consider the minimum heparin weight to be 2.3 kDa (Figure 5A), corresponding to 9 monosaccharides, and if we assume a linear length of 1 nm per disaccharide unit (Carlström, 1957), our results suggest that it is necessary to bridge intraprotein interactions (e.g., to create the opening from S to S^∗^) or interprotein interactions (e.g., to promote oligomerization; Ramachandran and Udgaonkar, 2011) across at least 4.5 nm distance to generate aggregation-prone species.

**FIGURE 8:**
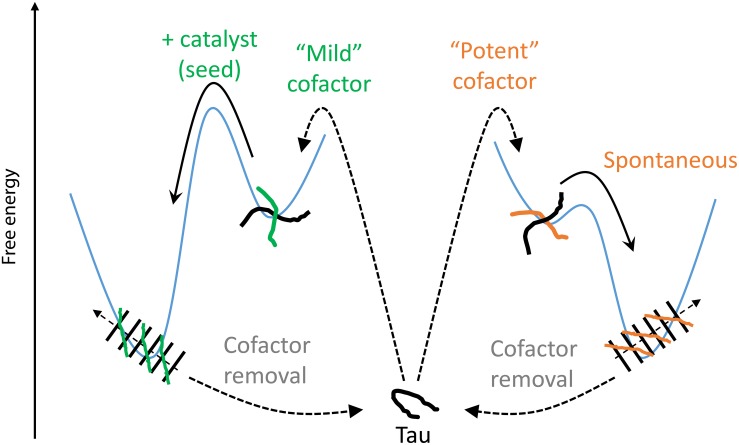
Thermodynamic model of cofactor-induced aggregation and cofactor assisted seeding. Mild cofactors (e.g., polyU) form metastable complexes that do not evolve to β-sheet containing species due to a high energy barrier. This barrier can be overcome by a catalyst such as a seed. In contrast, potent cofactors (e.g., heparin) form intermediate complexes, or oligomers, that spontaneously evolved toward amyloid fibrils. In both cases, removal of the cofactors from the mature fibrils results in their depolymerization, showing that the tau-cofactor complex, and not tau only, is the building block of these fibrils.

We introduce here the idea of “potent” and “mild” cofactors. Potent cofactors create unstable intermediates, such as those identified by EPR (Figure 4) and elsewhere in the literature (Shammas et al., 2015; Kjaergaard et al., 2018), which spontaneously convert to fibrils, given a low energy barrier to fibrils (Figure 8). The most prominent example of a potent cofactor is heparin that robustly triggers tau aggregation in a broad range of conditions. Mild cofactors create metastable complexes, which in contrast cannot spontaneously transform into fibrils given a considerable energy barrier to fibrils (Figure 8). Here we identified polyU RNA as a mild cofactor for tau187, which necessitates either a seed to overcome the energy barrier or an aggregation-prone tau mutant to lower the complex-to-fibril formation barrier. The observation that P301L facilitate the complex-to-fibril transition is in good agreement with early reports that P301L does not significantly increase the β-sheet structure in tau monomers, but rather increase the β-sheet formation upon cofactor addition (von Bergen et al., 2001; Fischer et al., 2007). Because this barrier crossing event is modulated by the nature of both partners, other RNA molecules or experimental conditions might trigger spontaneous aggregation of wild type tau (Kampers et al., 1996). Other mild cofactors are likely numerous under *in vivo* environments, but might have been overlooked because they do not spontaneously respond to commonly used ThT and TEM assays when mixed with tau. This model explains the underlying mechanisms by which RNA has been reported to allow efficient seeding (Meyer et al., 2014; Dinkel et al., 2015; Fichou et al., 2018a); RNA forms a metastable complex with tau that subsequently converts into fibrils in the presence of a seed.

This raises the following important question: in a cofactor-assisted experiment, are the properties of mature fibrils most influenced by the nature of the tau-cofactor complex (and therefore by the cofactor) or by the nature of the seed? This question is essential in the context of prion-like mechanisms (Sanders et al., 2014; Woerman et al., 2016), where one needs to understand the conditions required to ensure the faithful propagation of a given strain. Although cellular work has suggested that strain properties (based on proteolysis patterns and cellular localization of aggregates) can be maintained through seeding (Kaufman et al., 2016), other *in vitro* work suggested that properties of the seeded protein, and not the seed, dictate the structure of the obtained aggregates (Nizynski et al., 2018). Fichou and coworkers (Fichou et al., 2018a) showed that cofactor-assisted seeding achieve a mild degree of structural convergence compared to heterogeneous heparin fibrils, but without deciphering its origin. Recently, the structure of tau fibrils from CTE and CBD revealed the presence of a cofactor, whose identity however remains unknown, that is buried inside the tau aggregates (Falcon et al., 2019; Zhang et al., 2019b). Hence, it is reasonable to postulate that the cofactor-tau interaction would play a significant role in the final tau aggregate structure, in which the protein is “rolled” around the cofactor. In contrast, recently solved high-resolution structures of heparin fibrils (Zhang et al., 2019a) showed that heparin is not part of the core but might be scattered on the outside of the positively charged residues, suggesting that heparin might not be a critical determinant of the fibril morphology. This is consistent with the view that heparin increases the prevalence of aggregation-prone conformers (Figure 3) by exposing amyloidogenic segments, but does not specifically dictate the superstructural properties of the fibrils, yielding heterogenous fibrils (Fichou et al., 2018b; Zhang et al., 2019a).

Given the large structural diversity tau aggregates can adopt, we hypothesize that it is the interplay between the properties of (i) tau monomers (fragmentation, post-translational modifications, mutations…), (ii) the seed and (iii) the available cofactor (RNA, sterol, DNA…) that dictates the tau fold and packing that constitutes the structure of the final aggregate. In a cellular context, this hypothesis suggests that a given strain could be created/propagated when tau comes in contact with a specific cofactor [e.g., promoted by tau mislocalization (Thies and Mandelkow, 2007; Hoover et al., 2010) or abnormal homeostasis (Petrov et al., 2017)]. In addition, we highlighted here that tau-cofactor complexes can be isolated *in vitro*, and shown to only convert to fibrils when exposed to a potent seed. This suggests a possible cellular mechanism in which tau could associate with a locally upregulated cofactor, and remain inert until a competent seed is brought to its location, for instance through cell-to-cell propagation (Frost et al., 2009; Wang et al., 2017).

## Materials and Methods

### Protein Expression and Purification

A segment of tau, Figure 1, consisting of residues 255-441 was expressed in *E. coli* BL21 (DE3) and purified via nickel affinity chromatography as previously described (Pavlova et al., 2016). The mutation C291S was added by site directed mutagenesis and this construct is referred to as tau187 throughout the manuscript. Briefly, BL21 (DE3) cells transfected with a pET28a vector including the tau187 genes were grown in 1 L cultures of LB broth (Thermo Fisher Scientific) until an optical density of 0.6 A_600_ was achieved. Expression was induced through addition of 1 mM isopropyl β-D-1-thiogalactopyranoside (IPTG, Thermo Fischer Scientific) and incubated for 3 h. Cells were harvested by centrifugation at 6,000 × g (Beckman J-10) for 20 min. Purification was carried out by lysing the cells with 2 mg/ml lysozyme (Sigma-Aldrich) and pelleting cell debris through centrifugation. Subsequently, the supernatant was heated to 65°C for 12 min and unwanted proteins were precipitated and removed by centrifugation. Tau was extracted from the supernatant by binding to nickel affinity resin and subsequently removed by washing with increasing concentration of imidazole buffers. The protein was then buffer exchanged into the desired running buffer (20 mM ammonium acetate with 100 mM NaCl for experiments with heparin and with 0 mM NaCl for experiments with polyU, except when otherwise stated). The pH of the running buffer was 7.4 without protein and shifted to 7.8 with 100 μM tau187, due to poor buffering capacity of ammonium acetate. Several experiments were reproduced in 20 mM HEPES (which maintained pH at 7.4) to verify that the pH drift does not influence the conclusions of the manuscript (Supplementary Figure S16).

### Fibril Formation Using Heparin

Tau187 was incubated at 37°C with heparin at a tau:heparin molar ratio of 4:1 in 20 mM ammonium acetate and 100 mM NaCl, except when otherwise stated. Polydisperse heparin with an average molecular weight of 11 kDa (Sigma-Aldrich) was used, except when otherwise stated. Monodisperse purified heparin and desulfated heparin were bought from Galen Laboratory Supplies. When length of heparin was varied (Figure 5A), a tau:heparin mass ratio of 4:1 was used for each type of heparin. Spin-labeled tau187 was used in Figures 2 and 4 so that kinetics of ThT reading and EPR experiments could be directly compared. Spin-labeled tau187 was used in Figure 6B. Non-spin-labeled tau187 was used elsewhere.

### Fibril/Complex Formation Using PolyU

Tau187 was incubated with polyU (Sigma Aldrich, P9528; 10 μg/mL of polyU per 1 μM of tau187) in 20 mM ammonium acetate, except when otherwise stated. For Figure 6 and Supplementary Figures S11, S13, S14, S15, measurements were performed within 20 min after co-incubation of tau187 with polyU.

### ThT Fluorescence and Turbidity Measurement

ThT fluorescence intensity was measured with a Tecan M220 Infinite Pro plate reader for data in Figure 2 and Supplementary Figures S1, S2, S4, S20 and with a Biotek Synergy II for other data. A 20 μL sample volume was added to a Corning 384 Flat Black low volume well plate and covered with black vinyl electrical tape to prevent evaporation. For the Tecan plate reader, excitation wavelength of 450 nm (9 nm bandwidth) and emission wavelength of 484 nm (20 nm bandwidth) were used. Number of flashes was set to 25 and readings were taken from the bottom. For the Biotek plate reader, an excitation wavelength of 440 nm (30 nm bandwidth) and emission wavelength of 485 nm (20 nm bandwidth) were used. Number of flashes was set to 10 and readings were taken from the bottom. In the tau:heparin ratio study (Figures 2, 5C and Supplementary Figures S1, S3), the tau187 concentration was fixed to 100. For data in Figure 5, the concentrations of tau187 50 μM. For clarity, every 60th time points were shown in the ThT kinetic curves. In parallel of all fluorescence reading, absorption at 500 nm was recorded to verify the absence of liquid droplets (phase separation) in the initial conditions. 20 μM ThT was used for all fluoerescence experiments.

### Complex Aggregation Experiments (Figure 7)

After purification of the polyU + tau187 complex from Ni-affinity and subsequent concentration and buffer exchange, the protein concentration was estimated using spin-counting. The complex was diluted to reach 20 μM protein concentration and was mixed with 5% protein mass ratio of seeds. 20 μM ThT was used. The seeds consisted of tau187 mixed with heparin at a 4:1 tau:heparin molar ratio and incubated for 24 h at 37C. At 21 h, 25 μg/mL of RNase A was added in some of wells containing the complex + seed (labeled + RNase) while other wells received the same volume of buffer (labeled–RNase). The data presented in Figure 7A represents the ThT intensity measured from the given samples to which was subtracted the average intensity measured in 2 wells containing only buffer and 20 μM ThT. Raw data are presented in Supplementary Figure S18. TEM pictures in Figure 7 are obtained from a preparation of 80 μM tau187 and 0.8 mg/mL polyU after 10 min of incubation (“before seed”). 5% protein mass ratio of seeds was added to this same preparation for 24 h before taking the “24 h after seed” pictures.

### Spin Labeling

For experiments showed in Figures 2–4 and Supplementary Figure S4, tau187 was dissolved in 6 M guanidine hydrochloride and labeled by adding a 10-fold molar excess of spin label (1-oxyl-2,2,5,5,-tetramethylpyrroline-3-methyl) methanethiosulfonate (MTSL, Toronto Research Chemicals) or the diamagnetic analog of MTSL (1-Acetoxy-2,2,5,5,-tetramethyl-d-3-pyrroline-3-methyl) methanethiosulfonate (Toronto Research Chemicals), referred as dMTSL. For other experiments. Tau was reduced by addition of TCEP for at least 2 h. After removal of TCEP by PD-10 column, a 10-fold molar excess of MTSL (Toronto Research Chemicals) was added. The excess of spin labels was then removed by standard buffer exchange methods.

### Continuous Wave EPR

Continuous wave EPR measurements were acquired using a Bruker EMX X-Band spectrometer and dielectric (ER4123D) cavity. The microwave source applied ∼6 mW of power at 9.8 GHz using 0.3 G modulation amplitude and sweep width of 150 G. Samples of 3.5 μL volume were transferred to a 0.6 mm diameter quartz capillary and sealed with wax on both ends. A 2-D EPR spectrum was then acquired stepping through time. The concentration of MTSL labeled tau187 was 100 μM, and aggregation was induced with the appropriate molar ratio (4:1 tau:heparin) of 11 kDa Heparin (Sigma Aldrich) in phosphate final buffer (20 mM Sodium Phosphate, 100 mM Sodium Chloride, 100 μM EDTA, pH 7.0).

### Spin Counting

In order to obtain the protein concentration inside the complex, we used cw-EPR to measure the concentration of spin labels and infer the concentration of tau187. We measured the double integral of the acquired derivative spectra, which is proportional to the number of spins in the cavity. A 3^rd^ order polynomial fitted baseline was subtracted to the first integral before applying the second integration. The obtained double integral value was compared to the one from a radical (4-hydroxy-TEMPO) at known concentration to calculate the absolute concentration.

### Continuous Wave EPR Lineshape Analysis

EPR lineshape analysis was carried out as previously described (Pavlova et al., 2016) using the MultiComponent software developed by Dr. Christian Altenbach (University of California, Los Angeles), where a microscopic order macroscopic disorder (MOMD) model was used to describe the anisotropic motion of nitroxide radical (Hwang et al., 1975; Budil et al., 1996). The magnetic tensors A and g were fixed to those previously determined for MTSL spin labels attached to proteins (A_xx_ = 6.2 G, A_yy_ = 5.9 G, A_zz_ = 37.0 G, g_xx_ = 2.0078, g_yy_ = 2.0058, and g_zz_ = 2.0022) (Columbus et al., 2001).

In this study, the mobile component of this spin-labeled site was assumed to have isotropic motion with rotational diffusion tensor of R_x_ = R_y_ = R_z_, while the immobile and spin-exchanged components were assumed to have axially symmetric anisotropic motion. The spin-exchange component is subject to an ordering potential described by order parameter, S (Columbus et al., 2001; Pavlova et al., 2016). The number of fit parameters was kept at a minimum, which includes the population for each component (p), rotational diffusion constant (R), and order parameter (S). Rotational correlation time (t_R_) was calculated using t_R_ = 1/(6R).

The single-component fit was carried out from EPR spectra of tau monomer (without heparin addition) assuming the rotational diffusion without a restoring potential. For the three-component fit, in addition to mobile and immobile components, we included Heisenberg exchange frequency ω_ss_ in the third spin-exchanged component. Specifically, in order to fit the single-line, spin-exchanged component in the simulated EPR spectrum, the exchange frequency needs to be greater than the hyperfine interaction, i.e., > 100 MHz. To minimize the number of fitted parameters, we empirically optimized and used a fix value of ω_ss_ = 140 MHz in the simulation. We found that the three-component model using five fit parameters (p_1_, p_2_, t_R__1_, t_R__2_, S) obtained the best fit for the aggregated tau samples (Pavlova et al., 2016). The fit parameters used in Figure 2 are summarized in Supplementary Table S1.

### 3-Pulse ESEEM

ESEEM measurements were performed at X-Band on an ELEXSYS E580 spectrometer at 80 K with an Oxford instruments cryostat (CF935P) and temperature controller (ITC503). A Bruker MS3 resonator was maximally overcoupled to a Q-factor of approximately 100 for these measurements. A volume of 20–30 μL of sample was loaded into a 3 mm OD quartz EPR tube and flash frozen in liquid nitrogen before being inserted into the resonator. For each label position, both native cysteines were replaced by a serine while the specified residue number was replaced by a cysteine, which was subsequently labeled, i.e., the following tau187 mutants were measured: C322S/V309C, C322S/V313C, C322S/S316C and C322S/S404C. All experiments were carried out in deuterated phosphate final buffer and 30 wt% sucrose.

The pulse sequence for 3-pulse ESEEM (90°-τ-90°-T-90°-τ) consists of three 90°-pulses which generate a stimulated echo. When the delay between the 2nd and 3rd 90°-pulse is incremented, the amplitude of the stimulated echo is modulated at frequencies which correspond to the nuclear Larmor frequencies of nuclei coupled to the electron spin. The depth of the modulation is related to both the distance and number of nuclei around the electron spin (Erilov et al., 2005; Volkov et al., 2009). For our experiments, we specifically looked at deuterium nuclei in D_2_O. By deuterating the solvent water, we can distinguish between solvent nuclei and other nuclei in the sample. This allows us to selectively measure and quantify the water content at specific sites around tau protein.

For 3-pulse ESEEM measurements, the 90°-pulse length was 16 ns, and the delay, τ, between the 1st and 2nd 90°-pulses was set to 204 ns which suppresses ESEEM from proton nuclei. The delay between 2nd and 3rd 90-pulse was set to a starting value of 20 ns and was incremented by steps of 32 ns to a final value of 8160 ns.

The ESEEM data was processed by fitting the background decay to a polynomial (5th degree) and subtracting and dividing by the polynomial fit. The data was subsequently Hamming windowed, zero-filled to twice the original length and Fourier-transformed. The deuterium peak height in frequency domain is proportional to the total number of waters around the electron spin. For our analysis of the ESEEM data, we interpret the relative changes in deuterium peak height as changes in extent of hydration, i.e., large drops in relative peak height correspond to dehydration of the protein surface and indicate less solvent exposure. The raw data are shown in Supplementary Figures S21, S22.

### DEER

DEER measurement were carried out at Q-band (32 GHz) on an E580 pulsed EPR spectrometer (Bruker) at 50 or 80 K. Samples were prepared by mixing 50 μM of MTSL doubly labeled tau187 C322S/G272C/S285C at a 1:10 molar ratio with dMTSL labeled tau (500 μM) in 20 mM ammonium acetate buffer (20 mM ammonium acetate, 100 mM NaCl, 100 μM EDTA, pH = 7.0) supplemented with 30% sucrose. The samples were aggregated with appropriate molar ratio of heparin for 1 h at room temperature at which time they were flash frozen in liquid nitrogen. A dead-time free four-pulse DEER sequence was used. The pulse durations for the (π/2)_obs_, (π)_obs_, and (π)_pump_, were 22, 44, and 30 ns, respectively for the before, 4:1, 40:1, and 80:1 samples. The 8:1 and 20:1 samples utilized AWG capability with 32 ns Gaussian pulses. Distance distributions were determined using LongDistances.

### DLS

Dynamic light scattering (DLS) was performed on a Malvern Zetasizer Nano ZS. The samples were filtered at 0.22 μM before measurement. 100 μM tau187 and/or 1 mg/mL polyU were used in 20 mM ammonium acetate with 40 mM NaCl. For each sample, three consecutive measurements were acquired. At least three sample preparations were measured for each data set presented in Figure 6. Error bars represent the standard deviation among the different sample preparations. The autocorrelation functions were treated with automatic procedures implemented in Zetasizer software 7.10 assuming protein material and using the “general purpose” data analysis mode.

### Affinity Chromatography

Tau187 at 80 μM and/or polyU at 800 μg/mL were injected (250 μL injection volume) onto a Bio-Scale Mini Profinity IMAC Cartridge using a Biorad NGC liquid chromatography system. Samples were injected at 0.1 mL/min for 1 mL, before increasing the flow to 1 mL/min. The absorption at 280 nm was measured just after the column.

### Absorption Spectroscopy

Absorption spectra (Supplementary Figure S12) were acquired in a plate reader Biotek Synergy II. For each sample, 80 μL of sample was placed into a UV transparent plate.

### TEM

TEM grids, FORMVAR Carbon film on 300 mesh copper (Electron Microscopy Sciences), were floated on 10 μL of the fixed sample for 1 min and then blotted on filter paper. The grid was touched to 10 μL of deionized water, blotted, touched to 10 μL of the stain solution, 2% Uranyl Acetate (Electron Microscopy Sciences), blotted and then floated on 10 μL of 2% Uranyl Acetate for 1 min before blotting and setting aside to dry for 24 h. Imaging was carried out at room temperature using a JEOL JEM-1230 TEM at the UCSB NRI-MCDB Microscopy facility. Pictures of oligomers were obtained by incubating tau187 with poly U for 10 min at room temperature before preparing the grids.

## Data Availability Statement

The datasets generated for this study are available on request to the corresponding author.

## Author Contributions

YF, ZO, and SH prepared the manuscript and coordinated the experiments. C-YC carried out the EPR data acquisition and analysis, and aided in the preparation of figures. TK performed the ESEEM experiments and analysis, and aided in the interpretation and figure preparation. NE coordinated the DEER experiments and carried out the data analysis and figure preparation. ZO, YF, and HN handled the ThT experiments and data. HN and YF carried out the complex characterization by DLS and chromatography. YF, HN, ZO, and NE generated, expressed, and purified the protein mutants. All authors reviewed and approved the final manuscript.

## Conflict of Interest

The authors declare that the research was conducted in the absence of any commercial or financial relationships that could be construed as a potential conflict of interest.
