# Supplementary material for: Tau-Cofactor Complexes as Building Blocks of Tau Fibrils
Source: Front Neurosci. 2019 Dec 13;13:1339. doi: 10.3389/fnins.2019.01339 (PMC6923735; doi:10.3389/fnins.2019.01339)
Supplement: Supplementary file 1 [file Table_1.docx]

Supplementary Material

# Supplementary Experimental Methods

## Sonication Experiments

Spin-labeled tau187 was incubated for 24 h with heparin at a 4:1 or 80:1 tau:heparin molar ratio. An aliquot was removed as a control, and the remaining volume was sonicated for a total processing time of 2 min at 30% amplitude. Sonication pulses were alternating 4 s on with 3 s off and applied using a Misonex Ultrasonic Processor. Three aliquots were then removed and fluorescence was measured as described.

## Mixing Experiments

Spin-labeled tau187 was incubated with 11 kDa heparin at a 4:1 tau:heparin molar ratio. At 5 min, 2 hr and 24 hr time points, aliquots were removed and flash frozen in liquid nitrogen to serve as seed stocks. Seed stocks were thawed on ice and then mixed in equal volume with a monomer tau stock comprised of tau and ThT such that the total tau and ThT concentrations were unchanged after mixing. Fluorescence was measured.

## Expression of tau 2N4R

Tau isoform 2N4R carrying the mutations C322S and C291S was produced following the same expression and purification protocols used for tau187, described in the method of the main manuscript.

# Supplemental Figures

**
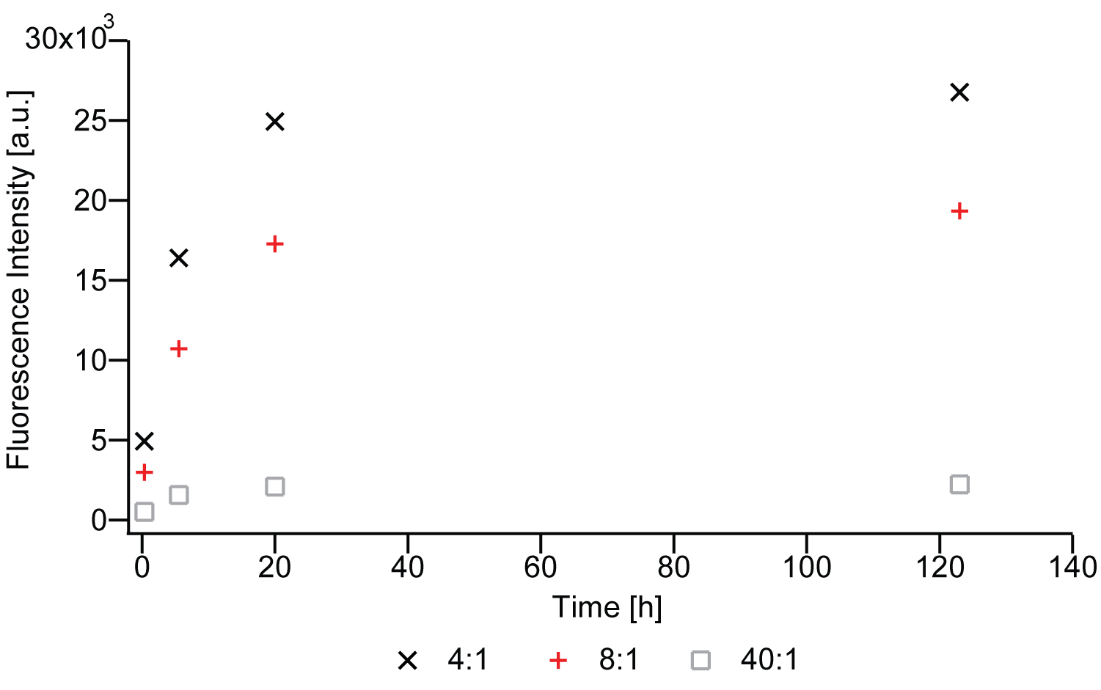
**

Figure S1. ThT Fluorescence verifying heparin stoichiometry trend in the absence of spin label (replicate of the main figure 2, but in the absence of spin label). Tau187 was aggregated with varying tau:heparin stoichiometric ratios. The stoichiometric trend is reproduced even in the absence of spin labels, i.e. lower heparin concentrations produce less total β-sheet content as measured by ThT. The data represents the average of five replicate trials and maximal standard deviation across all samples was ±612 fluorescence units which is approximately the size of the data markers.


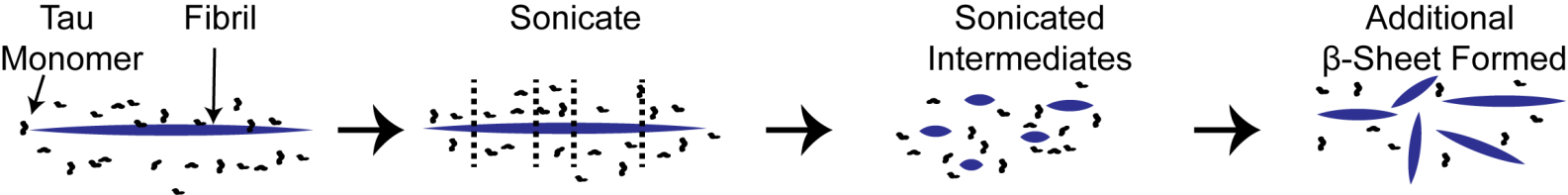


Figure S2. Hypothetical process by which sonication may increase total fibril content by providing more fibril ends for monomer addition. Sonicating fibrils should break down mature fibrils and increase the number of fibril ends for addition of new monomeric tau.


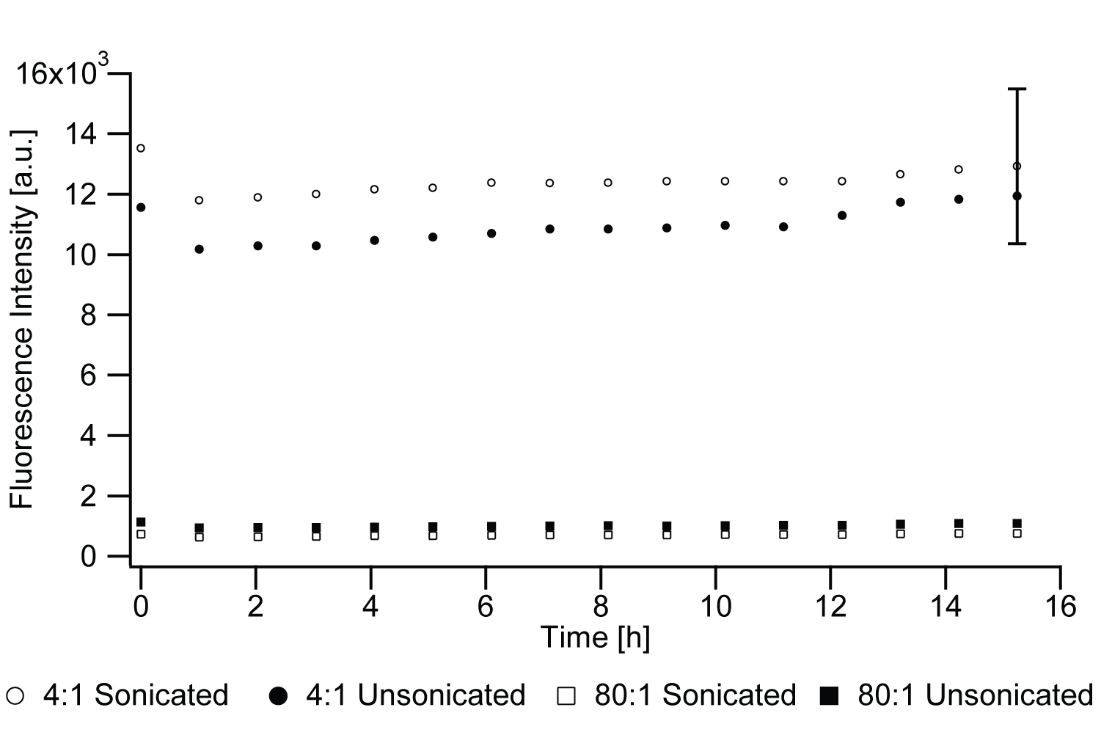


Figure S3. Sonication does not increase the total amount of fibrils, invalidating the hypothesis from figure S2. Mature tau187 fibrils (incubated for 24 hr with 80:1 or 4:1 tau:heparin ratio) were sonicated for 2 min before ThT fluorescence measurements. No significant difference was observed in ThT fluorescence, showing that increasing the number of fibril ends does not increase the total amount of cross-β sheets. Sonication efficacy at breaking fibrils was verified by TEM (figure S19). Standard deviation was calculated from triplicate experiments. Error bars are shown only on the final data point for clarity. Error bars on 80:1 samples are approximately the size of data markers.

**
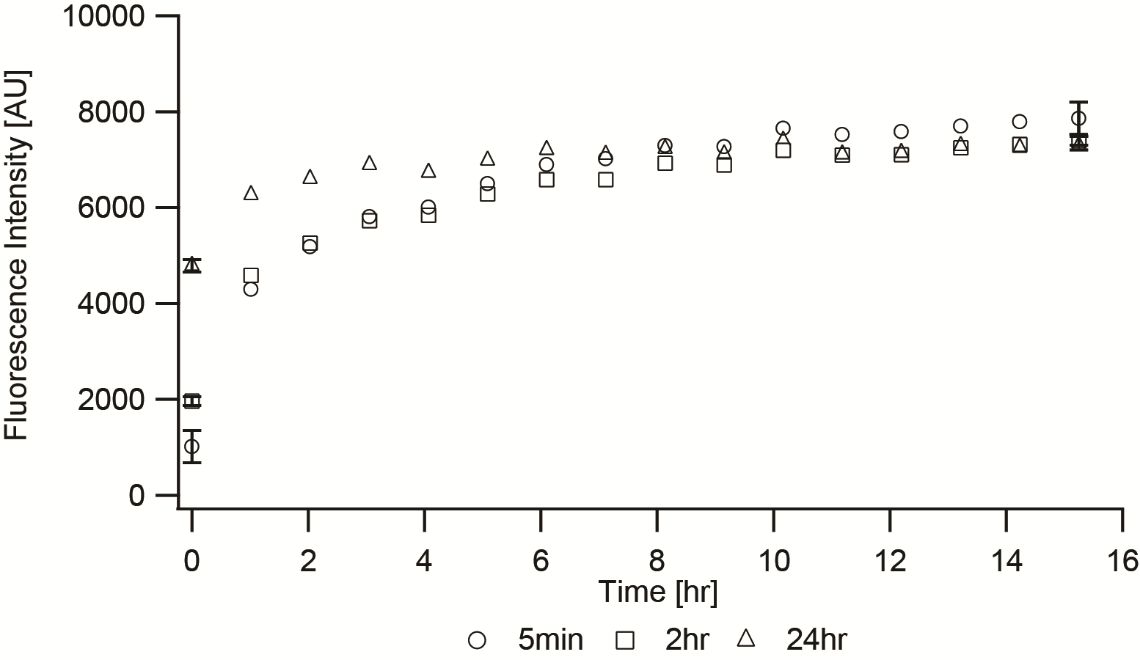
**

Figure S4. ThT fluorescence trace of pre-aggregated tau mixed with monomeric tau. Spin-labelled tau187 was incubated with heparin and aliquots were removed at 5 min, 2 hr and 24 hr after heparin addition. Each aliquot of pre-aggregated tau was mixed with an equal amount of monomer tau (no heparin). Time=0 represents the point of mixing the pre-aggregated tau with the monomer tau. Experiments were run in triplicate and standard deviation is shown only on the first and last data points for clarity. Despite differences at early times (originating from the mixing of different tau species), the final β-sheet content is similar. These results indicate no tau species are capable of overriding heparin and amplifying the total amount of β-sheets. The same experiment carried out with non-spin-labelled tau187 gave the same results (figure S20).


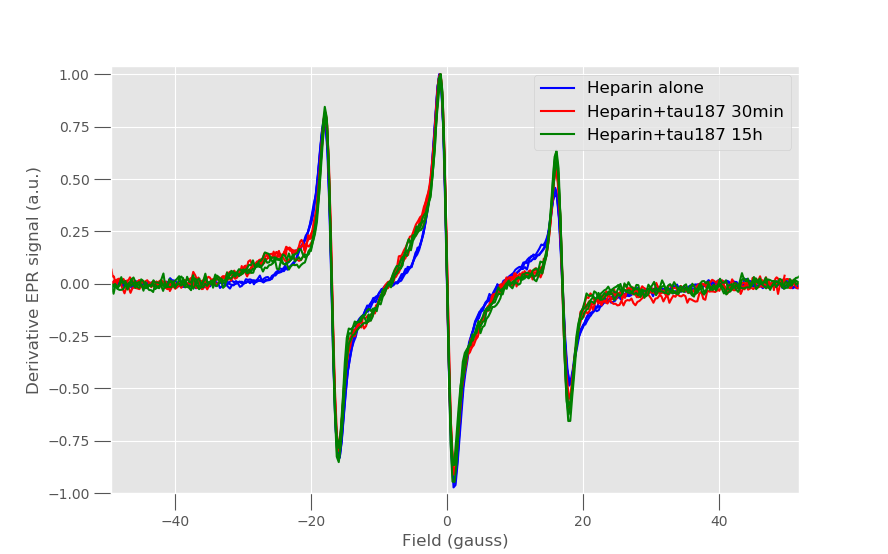


Figure S5: Cw-EPR lineshape of spin labelled heparin before (blue), after 30 min (red) and after 15 hr (green) incubation with tau187. The lineshape broadening (most likely due to weak spin exchange or/and lower correlation time**)** in the presence of tau187 shows a direct interaction between tau187 and heparin already after 30 min of incubation. Each condition was measured three times independently and the corresponding curves are overlaid with the same color.


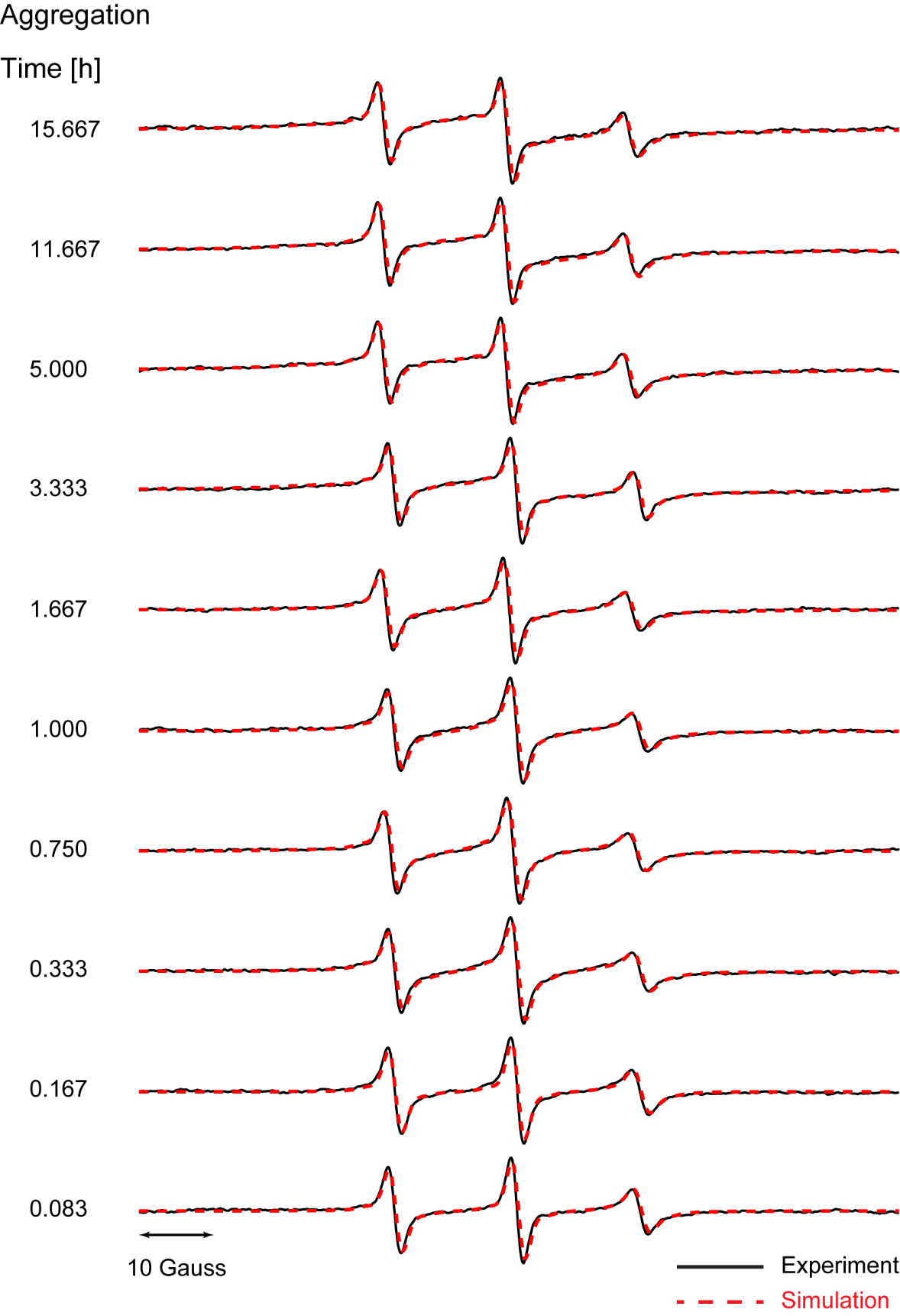


Figure S6. Cw-EPR spectra from EPR time course shown in figure 4. Simulation results (red) from three-component fits are overlaid onto experimental spectra (black). Summary of fit parameters are given in table S-1.


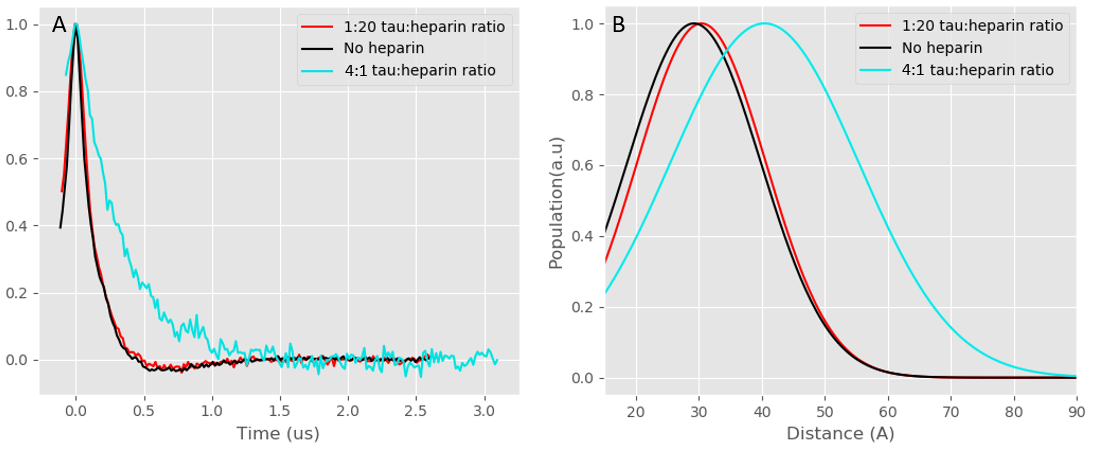


Figure S7: DEER dipolar evolution time after background correction (A) and fitted Gaussian distance distribution (B) for different tau:heparin ratios. DEER was measured 1 hr after addition of heparin. Data in panel B are duplicated from figure 5C inset.


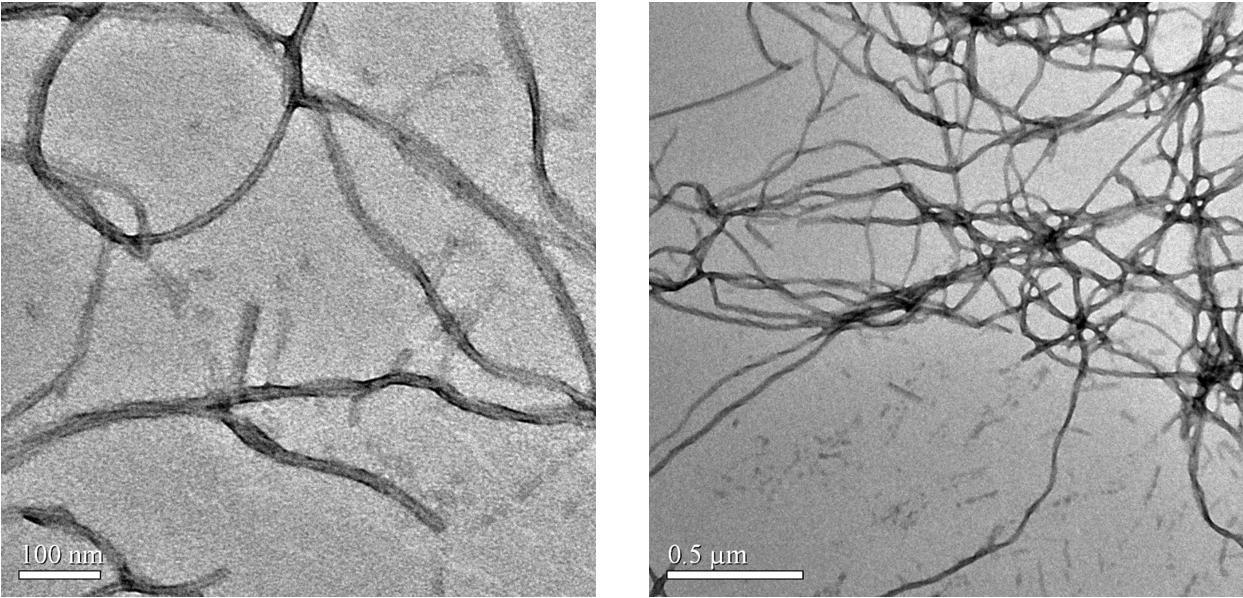


Figure S8. TEM pictures confirm that polyU incubated with tau187-P301L (ThT fluorescence shown in figure 5) spontaneously form fibrillary assemblies.


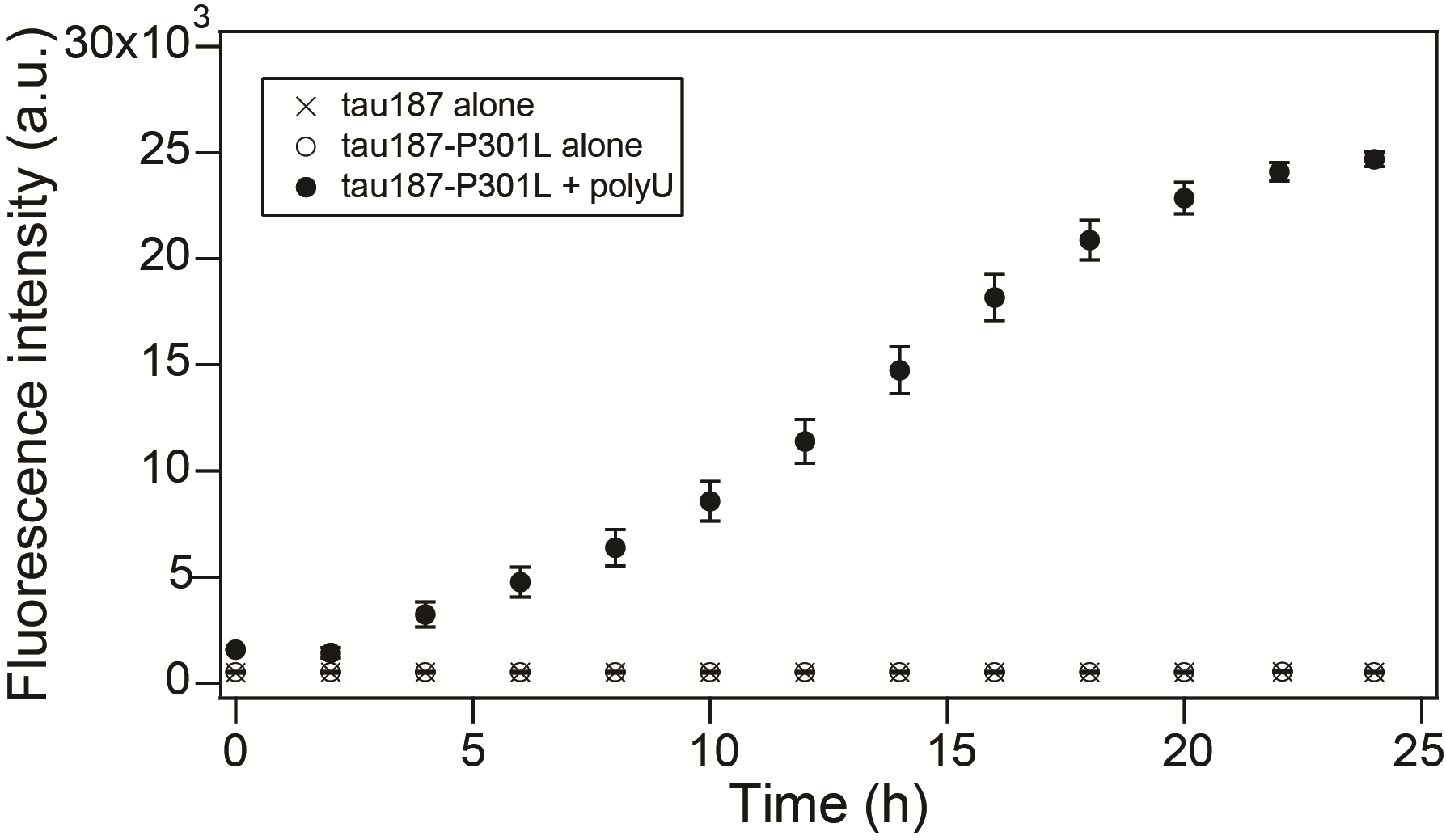


Figure S9. ThT fluorescence of tau187 and tau87-P301L. Neither of the two constructs aggregate at 37 C over 24 hr without cofactor. Protein concentration was 50 μM. Standard deviation was calculated from triplicate experiments.

**
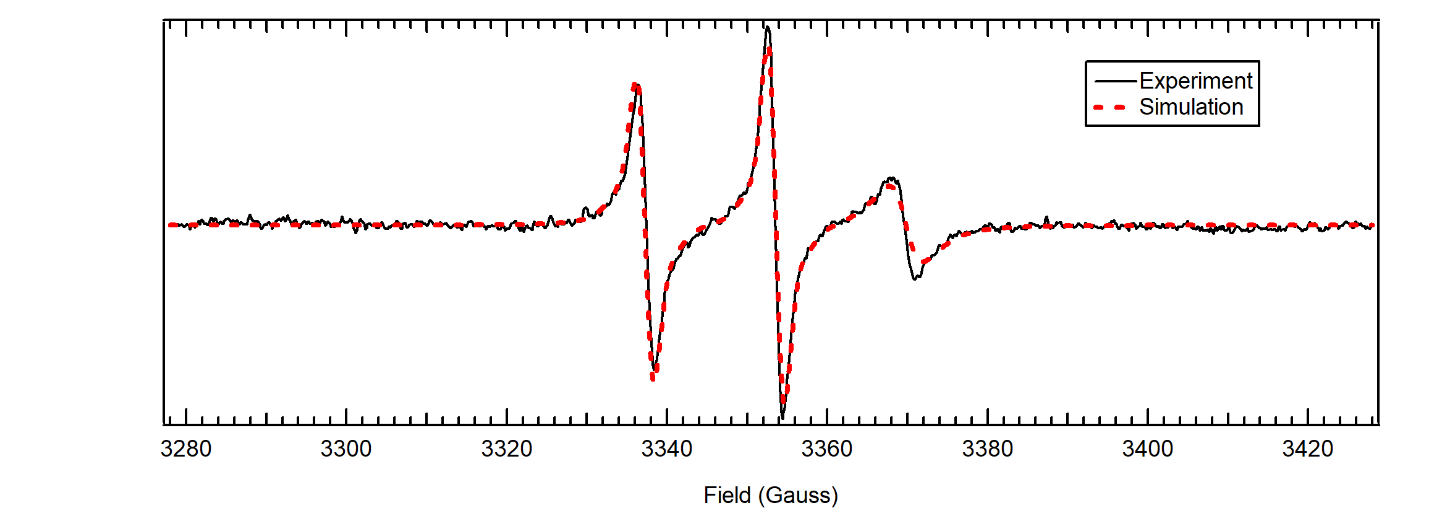
**

Figure S10. Cw-EPR spectrum of 100μM tau187 mixed with 1mg/ml polyU (black) and fitted with a single component (dashed red line) reaching a rotational correlation time of 1.3 ns.


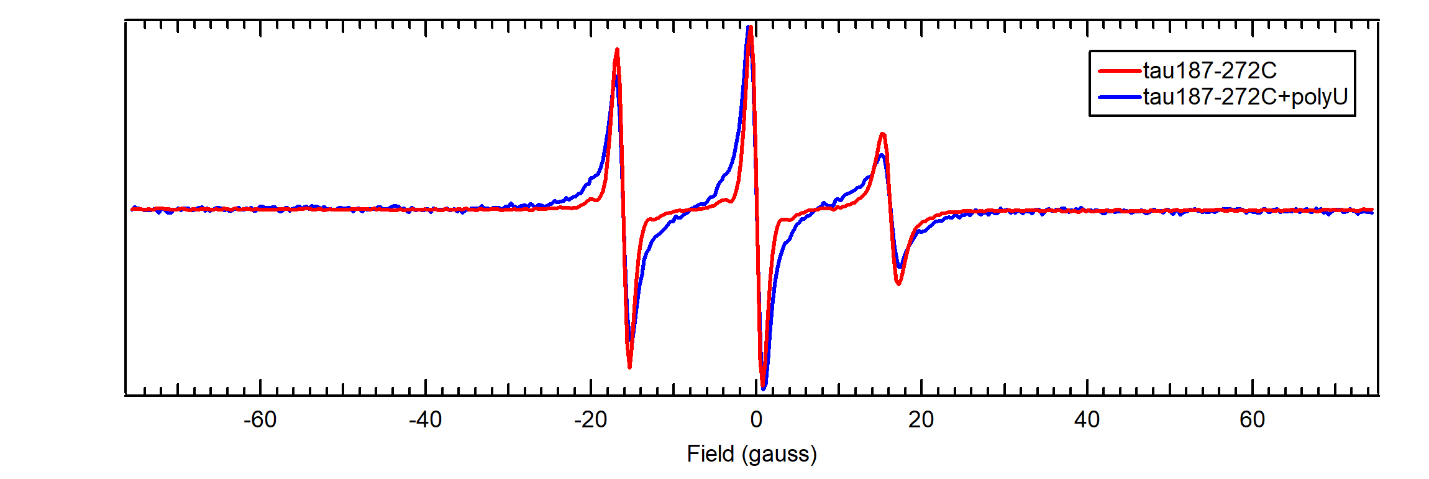


Figure S11. Cw-EPR spectra of 100 μM tau187 labelled at position 272 in the absence (red) and presence (blue) of 1 mg/mL polyU. The line broadening in the presence of polyU indicates a slower rotational time, similar to what was observed at position 322C (figure 6C).


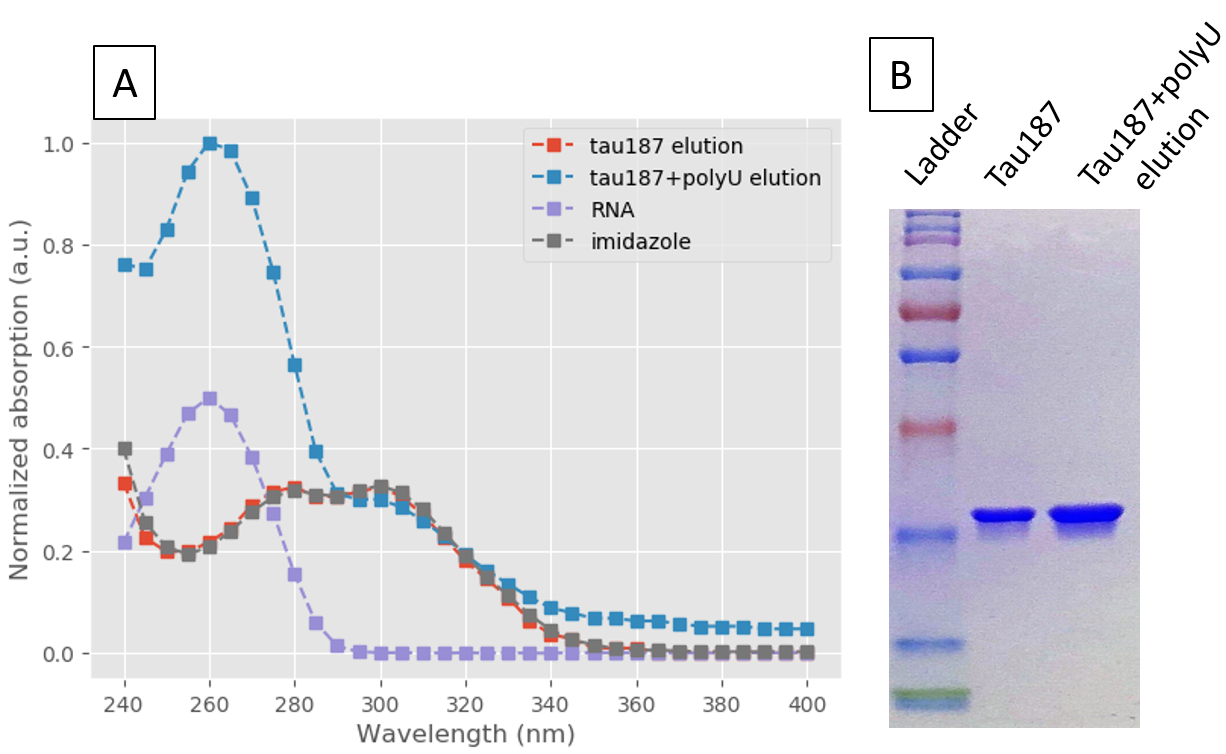


Figure S12. Elution peak from the Ni-affinity column (fraction 14 mL in figure 6B) analyzed by absorption spectroscopy (A) and SDS-page gel (B). When tau187+polyU is injected on the column, the elution fraction contains tau187 as shown on the SDS-page (B) and polyU as shown by absorption (A). The absorption spectra of tau187+polyU elution (blue) is the sum of the polyU spectrum (purple) and imidazole spectrum (grey, used as eluent). The spectral overlap of the imidazole and the elution from tau187-only injection (red) shows that the protein has no contribution to the observed absorption presented on the FPLC profile (figure 6B) due to a low absorption coefficient. The ladder on the left lane of the SDS-page gel has the following bands, from bottom to up: 4.6, 10 (green), 17, 26, 42 (red), 55, 72 (red), 95, 140 (red), 180, 250 kDa.


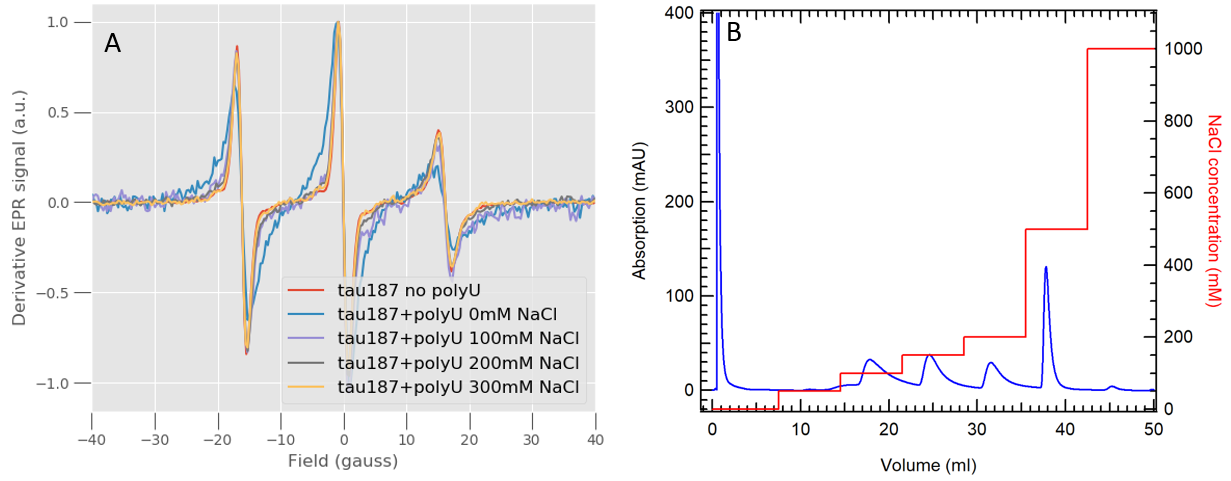


Figure S13. Tau187-polyU complex is electrostatically stabilized, as shown by cw-EPR (A) and affinity chromatography (B). The lineshape broadens from tau187 alone (red) to tau187+polyU at 0 mM NaCl (blue), indicating the formation of a complex. When increasing the salt concentration, the lineshape narrows and becomes, at 300 mM NaCl, indiscernible from the tau187 monomer. B) After immobilization of the complex on a Ni-affinity column, salt is progressively increased (red trace). The UV absorption (blue trace) shows that polyU is removed from the complex (i.e. detaches from tau that remains immobilized in the column) as salt is increased. All the polyU is removed between 200 and 500 mM. Note that there is about 3 mL delay between salt command (visible in the red trace) and the sensor cell (blue trace) due to tubing and pump volumes.

**
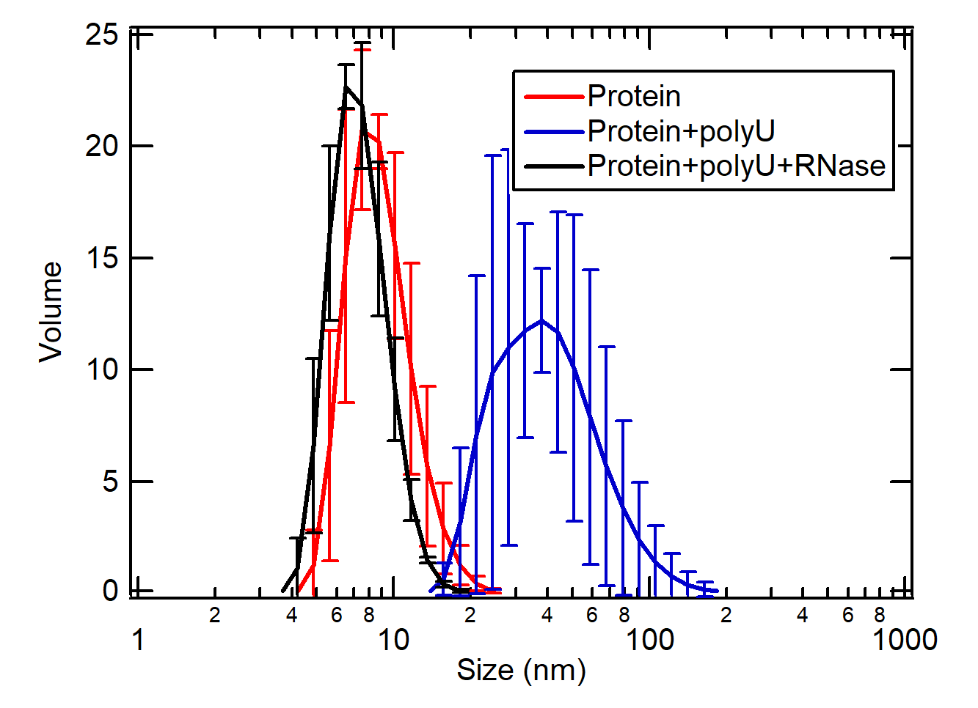
**

Figure S14. Hydrodynamic radius measured by DLS of tau187 (red, duplicated from figure 6A), tau187 mixed with polyU before (blue, duplicated from figure 6A) and 20min after adding RNase A (black). Addition of 25 μg/mL RNase destroys the tau-RNA complex seen by DLS. Error bars represent standard deviation over three repeats, each composed of 3 measurements of the same sample. Concentrations of tau187 and polyU were 100 μM and 1mg/mL, respectively.


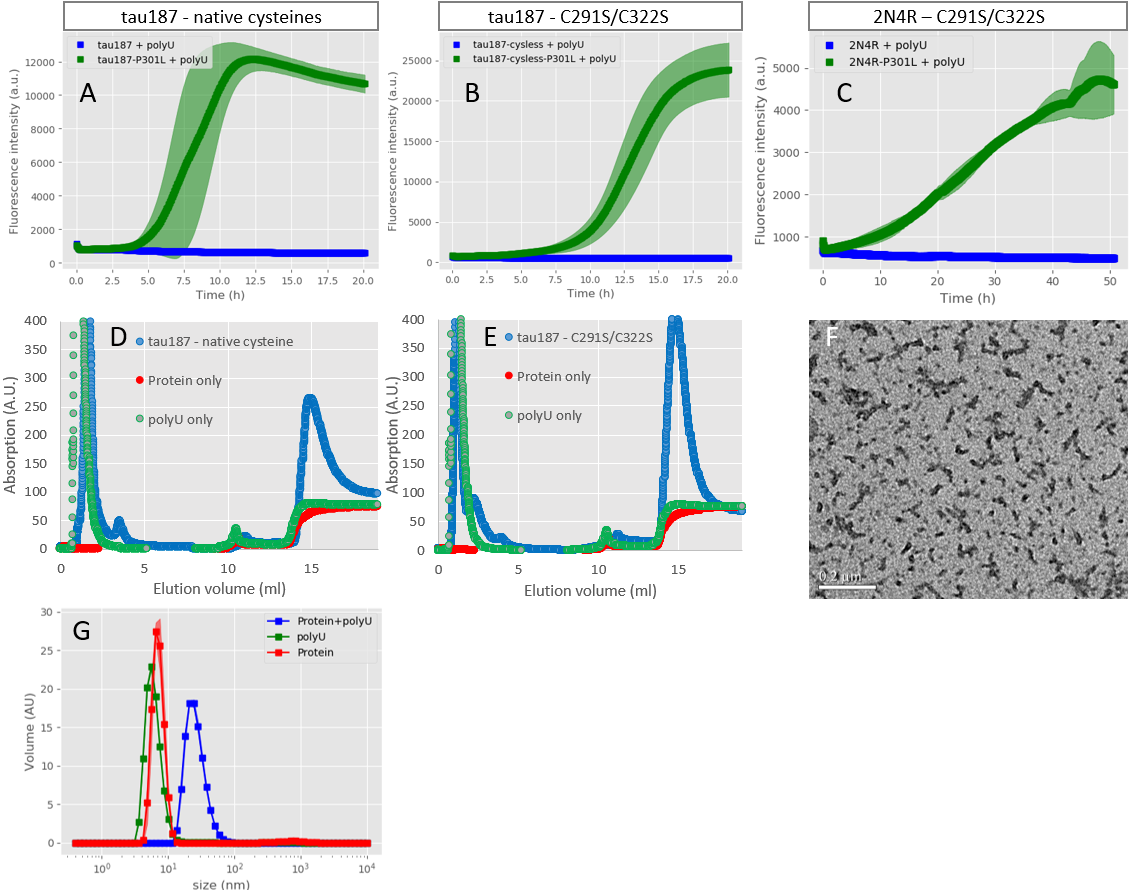
Figure S15: Validation of the results across different tau mutants and constructs: tau187 with both native cysteines (A, D and G), without any of the two native cysteines (B and E) and tau full length 2N4R without cysteines (C and F). ThT fluorescence (A, B and C) was measured while incubating 20 μM of protein, with or without the P301L mutation, in the presence of 0.2 mg/ml of polyU. For all constructs, the P301L mutation enables RNA-induced aggregation. Without the P301L mutation, the presence of oligomers was verified by Ni-affinity chromatography (D and E, following same procedure as in figure 6B; red and green traces are reproduced from figure 6B), by TEM (F) and by DLS (G, following same procedure as in figure 6A). Filled areas in panels A, B and C represent standard deviation over three, three and two independent repeats, respectively. Filled area in panel G (about the size of the line) represents standard deviation over three consecutive measurements of the same sample.


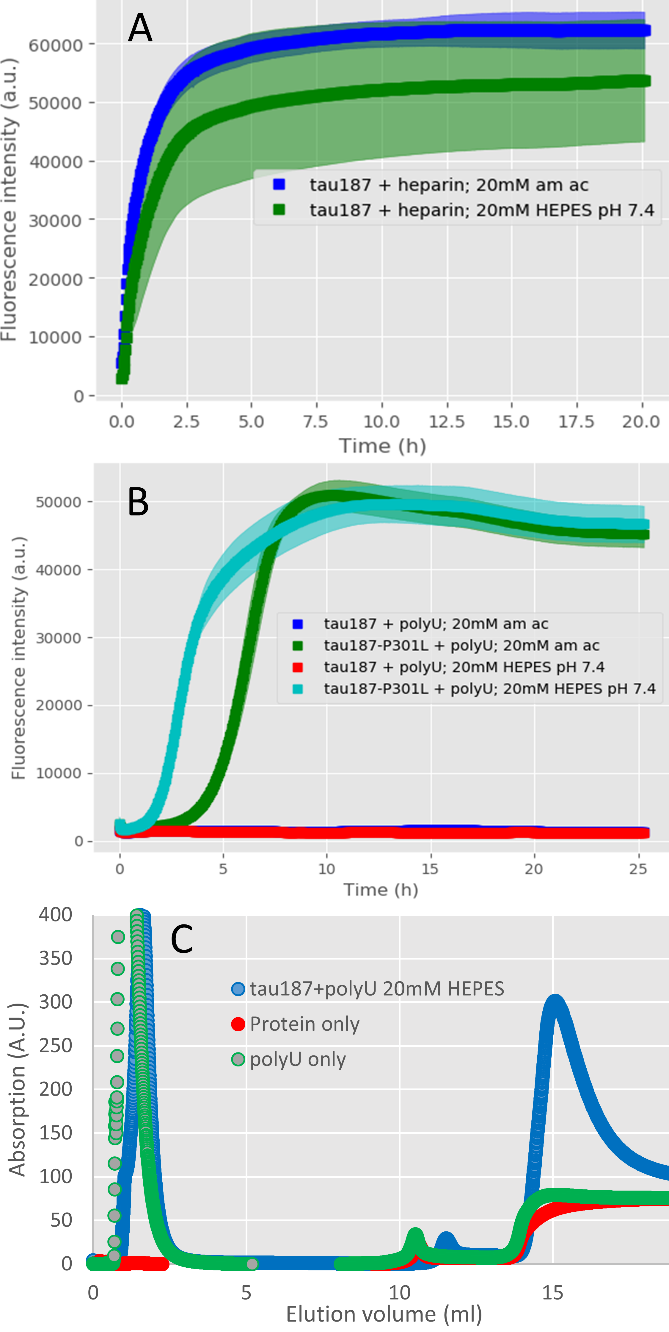


Figure S16: Comparison of two different buffers: 20 mM ammonium acetate, which pH drifts from 7.4 to 7.8 when adding 100 μM protein, and 20 mM HEPES at pH 7.4. A) Heparin-induced (4:1 tau187:heparin) aggregation is not significantly influenced by the buffer. B) polyU induces spontaneous aggregation only in the presence of P301L mutation. The difference in lag time between the buffers could be either associated with the different buffer nature or the pH drift of ammonium acetate. C) tau187-polyU complexes can be identified by Ni affinity chromatography. For A) and B), protein concentration was 100 μM, and the filled areas represent standard deviation over three independent repeats. In panel C, red and green are reproduced from figure 6 and injection conditions are as described in method section, except for the buffer.


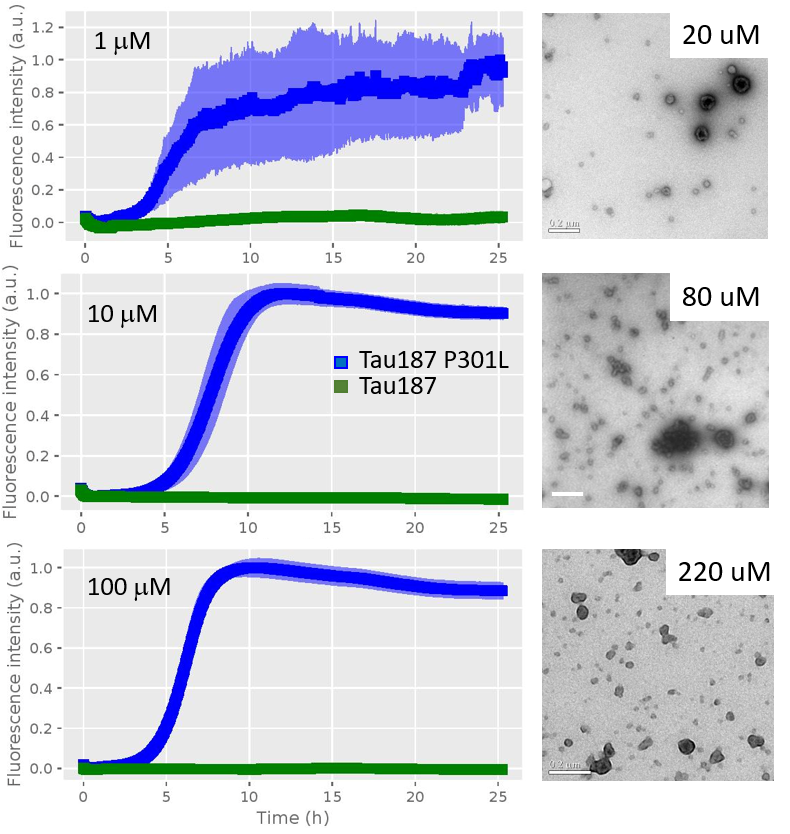


Figure S17: P301L enables spontaneous conversion of polyU-tau187 complexes in the range of 1-100 μM. Oligomers tau187-polyU are observed in the [tau] range 20-220 μM. Tau87:polyU ratio was 1 μM of tau for 10 μg/ml polyU in 20mM ammonium acetate. Filled areas in ThT curves represent standard deviation over 3 independent repeats.


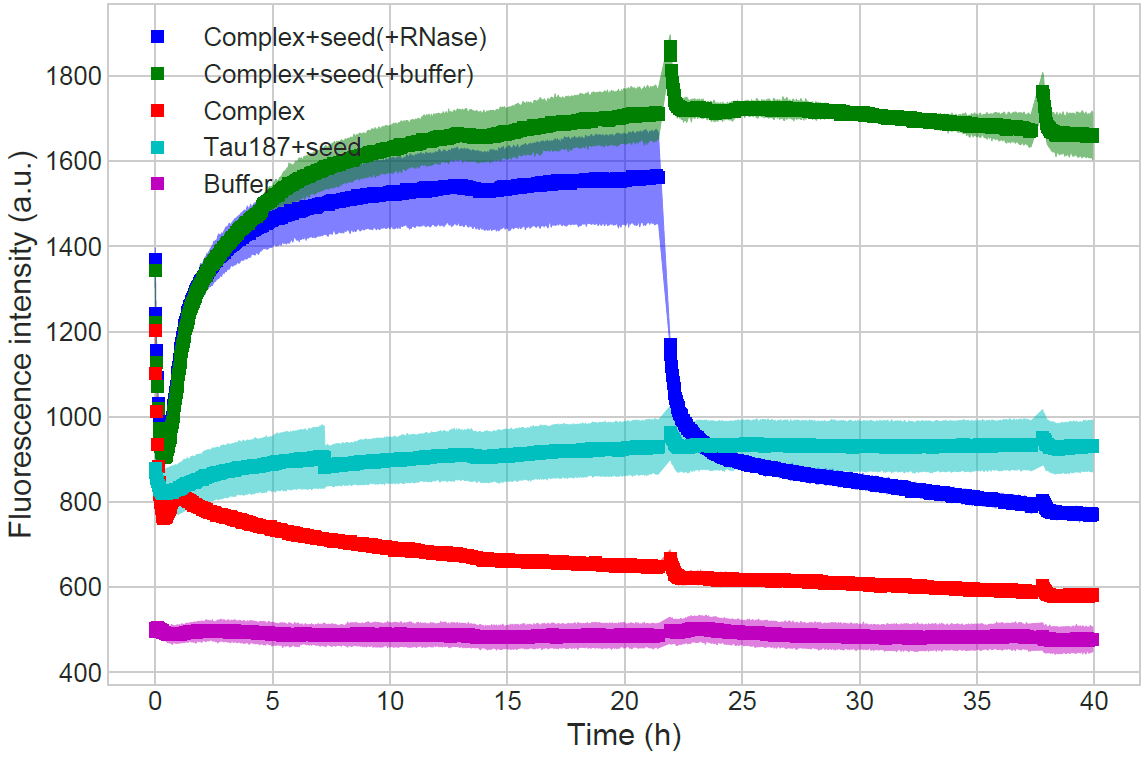


Figure S18. Full kinetics of complex seeding experiments reported in figure 7. The filled area is the standard deviation over 2 independent repeats (green and blue) or 3 independent repeats (cyan, red, purple).


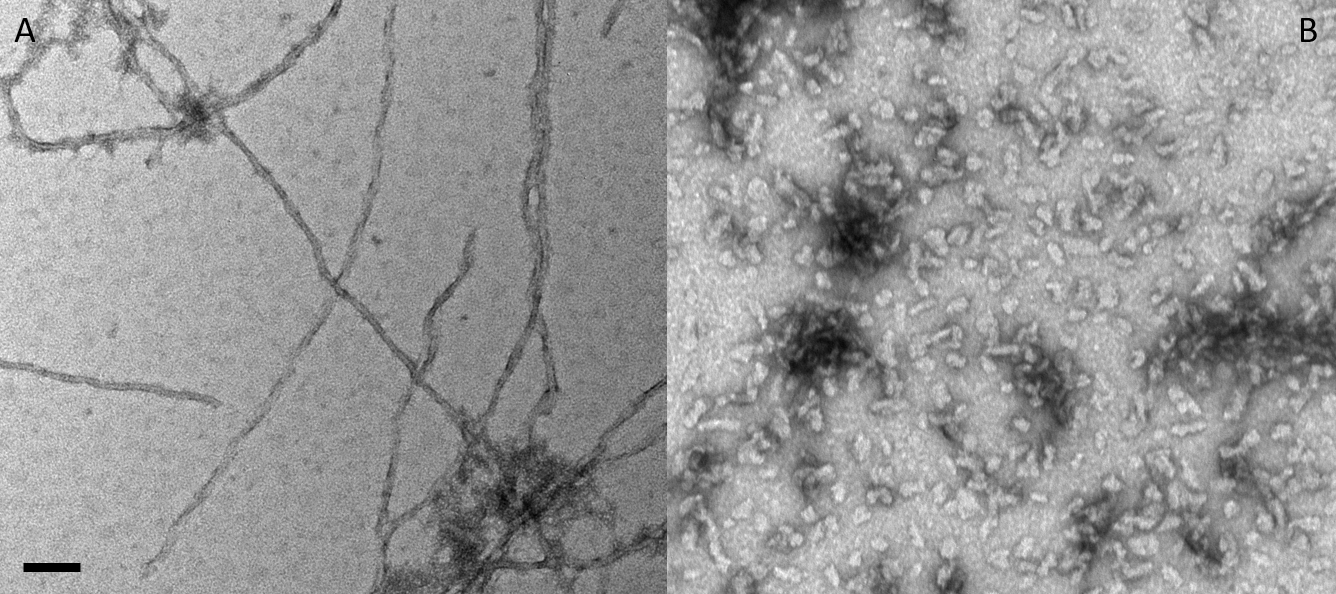


Figure S19. Verification by TEM of 2 min sonication efficacy at breaking fibrils. A) 4:1 tau187:heparin molar ratio before sonication B) 4:1 tau187:heparin molar ratio after sonication. Scale bar for both images is 100 nm.

**
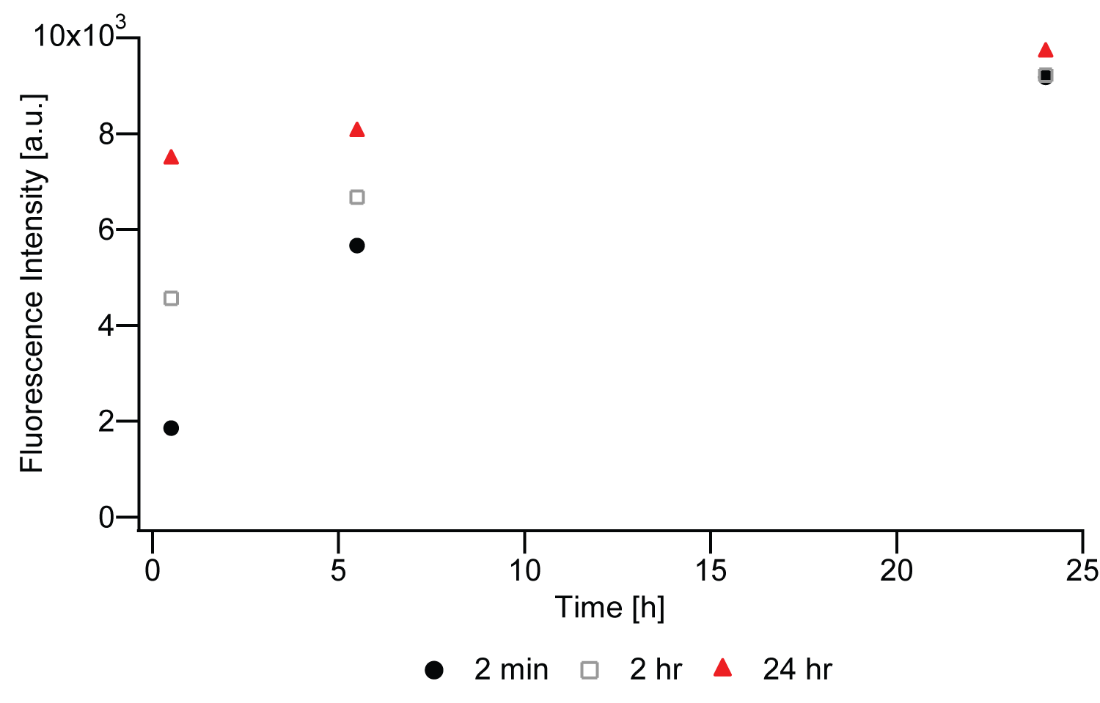
**

Figure S20. ThT fluorescence mixing experiment in the absence of MTSL spin labeling. The mixing experiment was carried out as described in figure S4. A convergence of total β-sheet content is observed independently of which pre-aggregated tau species are added, i.e. 5min, 2hr or 24hr. Experimental data is an average of six replicate trials and the maximum standard deviation across all samples was ±155 fluorescence units which is approximately the size of the data markers.


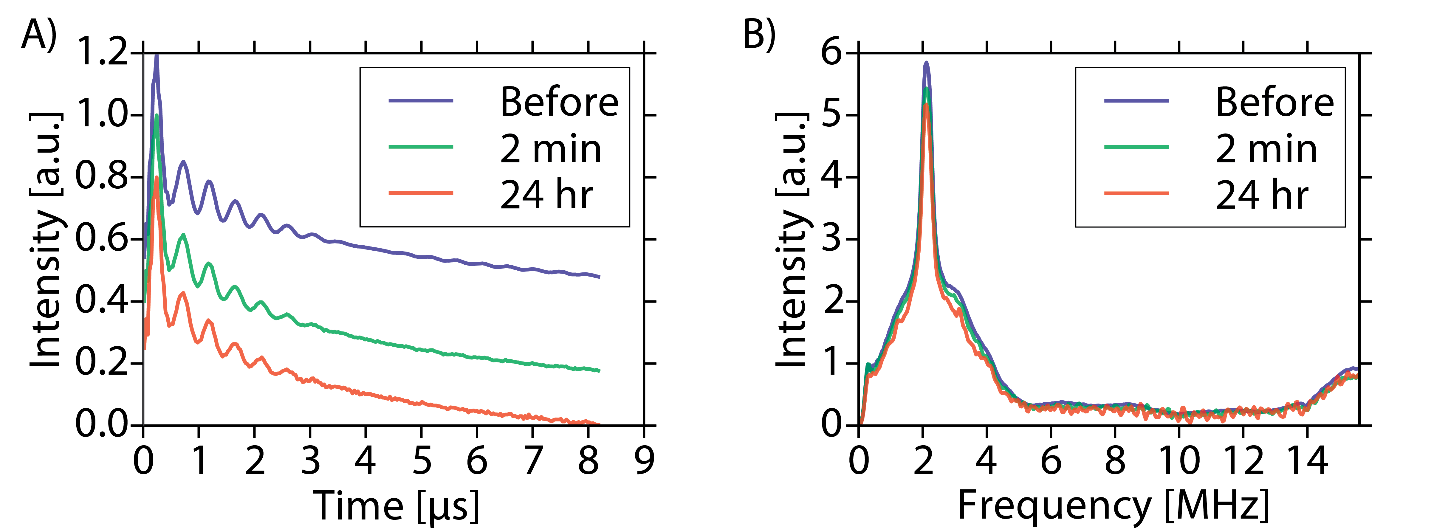


Figure S21. ESEEM data of labeled tau187 C322. A) Raw ESEEM time trace before heparin addition, 2 min and 24 h after heparin addition data. B) The respective ESEEM data shown in A processed and Fourier transformed as described in Supplementary Methods. The narrow peak at 2.12 MHz is due to matrix deuterium nuclei in D_2_O which are > 3 Å from the electron spin. The broader peak centered around the same frequency is due to hydrogen-bonded deuterium nuclei. The amplitude of the peak at ~2 MHz is therefore the contribution of both matrix and hydrogen bonded deuterium nuclei and reflects the relative hydration when we compare different sites and aggregation times.


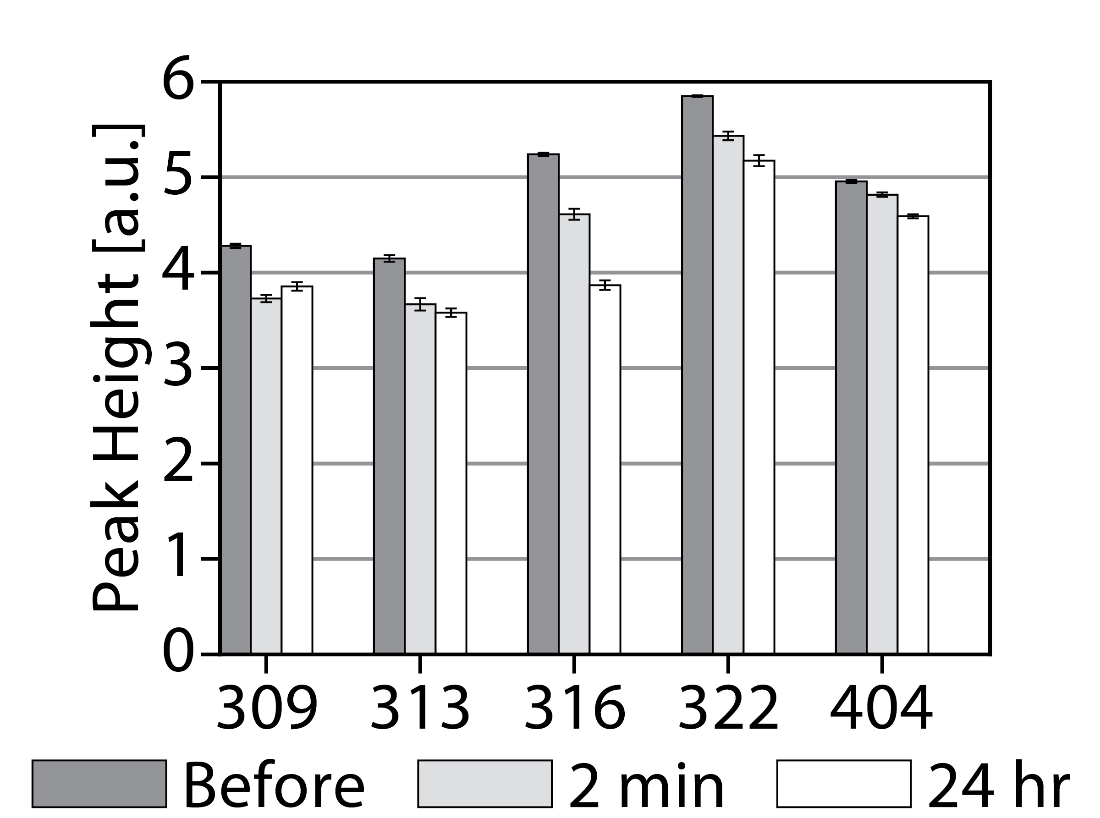


Figure S22. The ESEEM data shown in figure 4 are normalized. Shown here is the raw peak intensities prior to normalization by the before aggregation peak height.

**Table S-1**

| Aggregation time (min) | Aggregation time (hr) | $p_{1}$(%) | $\tau_{R,1}$ (ns) | $p_{2}$(%) | $\tau_{R, 2}$ (ns) | $p_{3}$(%) | *S* |
| --- | --- | --- | --- | --- | --- | --- | --- |
| 0 | 0 | 100.0 | 1.1 | 0 | 3.6 | 0 | 0.62 |
| 5 | 0.083 | 48.4 | 1.0 | 44.3 | 3.6 | 7.3 | 0.62 |
| 10 | 0.167 | 45.4 | 1.0 | 42.3 | 3.7 | 12.3 | 0.64 |
| 20 | 0.333 | 43.6 | 1.0 | 40.8 | 3.4 | 15.6 | 0.65 |
| 45 | 0.750 | 39.3 | 1.0 | 38.2 | 3.5 | 22.5 | 0.66 |
| 60 | 1.000 | 32.8 | 1.1 | 34.8 | 3.4 | 32.4 | 0.65 |
| 70 | 1.167 | 32.3 | 0.9 | 26.6 | 3.2 | 41.4 | 0.64 |
| 80 | 1.333 | 31.5 | 0.9 | 23.9 | 3.0 | 44.6 | 0.63 |
| 100 | 1.667 | 30.5 | 0.9 | 18.1 | 2.9 | 51.6 | 0.62 |
| 200 | 3.333 | 26.1 | 0.9 | 17.3 | 2.8 | 56.6 | 0.63 |
| 300 | 5.000 | 24.4 | 0.9 | 10.9 | 2.6 | 64.7 | 0.61 |
| 500 | 8.333 | 20.6 | 0.8 | 12.4 | 2.6 | 67.0 | 0.62 |
| 700 | 11.667 | 21.2 | 0.8 | 12.2 | 2.5 | 66.6 | 0.62 |
| 800 | 13.333 | 16.5 | 0.8 | 13.9 | 2.6 | 69.6 | 0.62 |
| 940 | 15.667 | 17.3 | 0.8 | 14.6 | 2.7 | 68.1 | 0.62 |

Table S-1. Summary of calculated fit parameters for EPR line shape simulation (see corresponding EPR spectra figure S6). Shown are calculated population distributions p_1_ (mobile), p_2_ (immobile) and p_3_ (β-sheet). Collectively, p_2_ and p_3_ are referred to as the interfacial population. τ_R,1_ and τ_R,2_ are the respective rotational correlation times and S is the order parameter.

**References**

Erilov, D. A., Bartucci, R., Guzzi, R., Shubin, A. A., Maryasov, A. G., Marsh, D., et al. (2005). Water concentration profiles in membranes measured by ESEEM of spin-labeled lipids. *J Phys Chem B* 109, 12003–12013. doi:10.1021/jp050886z.

Margittai, M., and Langen, R. (2006). Side Chain-dependent Stacking Modulates Tau Filament Structure. *Journal of Biological Chemistry* 281, 37820–37827. doi:10.1074/jbc.M605336200.

Milov, A. D., Samoilova, R. I., Shubin, A. A., Grishin, Yu. A., and Dzuba, S. A. (2008). ESEEM Measurements of Local Water Concentration in D2O-Containing Spin-Labeled Systems. *Appl Magn Reson* 35, 73–94. doi:10.1007/s00723-008-0144-2.

Volkov, A., Dockter, C., Bund, T., Paulsen, H., and Jeschke, G. (2009). Pulsed EPR Determination of Water Accessibility to Spin-Labeled Amino Acid Residues in LHCIIb. *Biophys J* 96, 1124–1141. doi:10.1016/j.bpj.2008.09.047.

Zhang, W., Falcon, B., Murzin, A. G., Fan, J., Crowther, R. A., Goedert, M., et al. (2019). Heparin-induced tau filaments are polymorphic and differ from those in Alzheimer’s and Pick’s diseases. *eLife* 8, e43584. doi:10.7554/eLife.43584.
